# Marker-Free Genome Engineering in *Amycolatopsis* Using the pSAM2 Site-Specific Recombination System

**DOI:** 10.3390/microorganisms10040828

**Published:** 2022-04-16

**Authors:** Luísa D. F. Santos, Laëtitia Caraty-Philippe, Emmanuelle Darbon, Jean-Luc Pernodet

**Affiliations:** Université Paris-Saclay, CEA, CNRS, Institute for Integrative Biology of the Cell (I2BC), 91198 Gif-sur-Yvette, France; luisa.ferreira-santos@i2bc.paris-saclay.fr (L.D.F.S.); laetitia.philippe@i2bc.paris-saclay.fr (L.C.-P.)

**Keywords:** *Amycolatopsis*, pSAM2 site-specific recombination system, excision tool, large-scale deletion, unmarked mutant, marker recycling

## Abstract

Actinobacteria of the genus *Amycolatopsis* are important for antibiotic production and other valuable biotechnological applications such as bioconversion or bioremediation. Despite their importance, tools and methods for their genetic manipulation are less developed than in other actinobacteria such as *Streptomyces*. We report here the use of the pSAM2 site-specific recombination system to delete antibiotic resistance cassettes used in gene replacement experiments or to create large genomic deletions. For this purpose, we constructed a shuttle vector, replicating in *Escherichia coli* and *Amycolatopsis*, expressing the integrase and the excisionase from the *Streptomyces* integrative and conjugative element pSAM2. These proteins are sufficient for site-specific recombination between the attachment sites *attL* and *attR*. We also constructed two plasmids, replicative in *E. coli* but not in *Amycolatopsis*, for the integration of the *attL* and *attR* sites on each side of a large region targeted for deletion. We exemplified the use of these tools in *Amycolatopsis mediterranei* by obtaining with high efficiency a marker-free deletion of one single gene in the rifamycin biosynthetic gene cluster or of the entire 90-kb cluster. These robust and simple tools enrich the toolbox for genome engineering in *Amycolatopsis*.

## 1. Introduction

Actinobacteria of the genus *Amycolatopsis* are the source of diverse bioactive specialized metabolites [1]. Some species such as *Amycolatopsis mediterranei* and *Amycolatopsis orientalis* are for instance used industrially for the production of the medically important antibiotics rifamycins [2] and vancomycin [3,4], respectively. *Amycolatopsis* spp. are also known for their biotechnological potential as agents of lignin degradation [5] and bioremediation [6] or for the fermentative production of aromatic compounds [7].

Despite the importance of the genus *Amycolatopsis*, the development of adapted genetic tools has lagged behind that for other actinobacterial genera such as *Streptomyces* [8,9]. For some time, the introduction of DNA into *Amycolatopsis* has been a limiting factor, until this problem could be circumvented by the development of a method of direct transformation of *Amycolatopsis* mycelia [10] or by using intergeneric conjugation from *Escherichia coli* to introduce single-stranded DNA into the *Amycolatopsis* host [11,12]. Functional studies often rely on the inactivation/deletion of one or several genes. Recent attempts to use CRISPR-Cas based systems in *Amycolatopsis* suggested a toxicity linked to dead *Cas9* expression [13]. Thus, a genome editing system based on another Cas protein was developed to obtain deletion mutants in *Amycolatopsis mediterranei* U32 [13], however this implicated the integration of the *Cas12a* gene and of a hygromycin resistance gene in the chromosome of *A. mediterranei* U32. To obtain unmarked gene replacement, a more common technique for gene deletion is often used. In this multi-step process, fragments identical to those upstream and downstream of the target gene are cloned contiguously into a suicide vector carrying a selectable marker. This delivery vector is introduced into the actinobacterial strain and its integration by one event of homologous recombination is selected. The transformants are grown for one or several rounds of cultivation under non-selective conditions and single colonies are then screened for the loss of the resistance marker carried by the vector. Sensitive clones, devoid of the vector, are either wild-type (if both homologous recombination events occurred within the same homologous fragment) or deleted of the target gene (if the first and second recombination events occurred within different homologous fragments). Wild-type and mutant colonies can be distinguished by screening, for example using PCR. To facilitate the obtention of mutants by this approach, various methods of counterselecting [14,15,16] the delivery vectors or of visual screening [17,18] have been used in *Streptomyces*. In *Amycolatopsis* a chromogenic screening method based on the *gusA* gene was developed, allowing to distinguish clones still carrying the suicide vector from those who have lost it [7]. A counterselection *rpsL*-based streptomycin resistance system was also developed for gene deletion in *Amycolatopsis* [8]. This system, based on the dominance of streptomycin sensitivity, can only be used in a streptomycin resistant mutant.

Other techniques, allowing marker free deletions and marker recycling, based on site-specific recombination (SSR) have been developed to remove the antibiotic marker after knockout mutant selection in some actinobacteria [19]. These techniques are based on the use of an antibiotic resistance gene cassette flanked by SSR sequences, which are recognized by cognate site-specific recombinases. The excision of the antibiotic cassette leaves a short scar (usually smaller than 50 bp) in the chromosome and can be designed to avoid polar effect [19]. Another application of SSR systems for genome engineering is the deletion of complete gene clusters or large genomic regions. Several examples of such application have been reported in *Streptomyces*, relying on the Cre/*loxP* SSR system (Herrmann et al. [20] and references therein). This approach allowed the deletion of genomic regions up to 1.5 Mb in *Streptomyces avermitilis* [21].

An example of well-characterized SSR system is that of pSAM2, an integrative element from *Streptomyces ambofaciens* [22]. The integrase encoded by the *int* gene promotes the integration of pSAM2 into the chromosome by intermolecular SSR between the attachment (*att*) sites, *attP* carried by pSAM2 and *attB* located in the bacterial chromosome. After integration, pSAM2 is flanked by the *attL* (left) and *attR* (right) sites. These sites can be themselves involved in intramolecular SSR leading to excision of pSAM2, an event requiring both the excisionase (encoded by the *xis* gene) and the integrase. Detailed studies have been carried out to precisely define the minimal sites required by the pSAM2 SSR system [23,24]. These findings were used for the construction of several excisable antibiotic resistance cassettes in which minimal *attL* and *attR* sites flank various antibiotic resistance genes [25]. These selectable cassettes are used in *Streptomyces* to replace target genes by homologous recombination. After positive selection of the mutant strains, the resistance cassette can be efficiently excised by transiently expressing *xis* and *int* carried by an unstable replicative vector. The resulting mutant strains are marker-free and contain a minimal *attB* site. The size of the scar (33, 34, or 35 bp) is dependent of the excisable cassette used and is chosen in order to maintain the correct reading frame if the deletion is internal to a coding sequence [25].

These cassettes allow marker recycling and could be useful in *Amycolatopsis* strains, many of which are naturally resistant to several antibiotics [26]. This limits the choice of resistance markers and makes the possibility of recycling them even more attractive. However, if these cassettes can be used for gene replacement in *Amycolatopsis*, the existing plasmids promoting their excision are unable to replicate in *Amycolatopsis*. The same is true for plasmids carrying other SSR systems that have been used in *Streptomyces*, as they all rely on replicons from pIJ101 or pSG5 [10].

Until now, few plasmids have been described in *Amycolatopsis* species [27], and only four of them (pMEA100, pMEA300, pA387 and pXL100) have been further studied. However, only the replicative vectors derived from the endogenous plasmids pA387 and pXL100 have been shown to replicate into several *Amycolatopsis* species [12,28].

Here, we report the successful use of the pSAM2 SSR system to construct unmarked deletion mutants in *Amycolatopsis*, without leaving exogenous sequences within the genome, except for a short scar of 33 bp. For this purpose, we constructed a shuttle vector, replicating in *Escherichia coli* and *Amycolatopsis*, which is poorly maintained in *Amycolatopsis* in the absence of a selection pressure. This plasmid, derived from pRL60 (containing the short pA387 replicon) [29], carries the *xis* and *int* genes from pSAM2 and the *oriT* origin of transfer for interspecific conjugation. Moreover, we used the pSAM2 SSR system for the generation of large deletions. For this purpose, we constructed two plasmids for the integration of the recombination sites *attL* and *attR* on each side of a region targeted for deletion. With these newly constructed tools and the previously constructed excisable antibiotic resistance cassettes [25] we demonstrated the efficiency of the pSAM2 SSR system to obtain marker-free in-frame deletion mutants and to generate large-scale deletions in *A. mediterranei* DSM 40773. These tools enrich the genetic toolbox available for genetic engineering of *Amycolatopsis* strains.

## 2. Materials and Methods

### 2.1. Bacterial Strains, Cultivation Conditions and Strain Manipulation

All strains used in this study are listed in Table 1. *E. coli* strains were grown at 37 °C in LB. When required, antibiotics were added to *E. coli* cultures in liquid (or on solid) medium at the following concentrations: ampicillin, 50 µg/mL (or 100 µg/mL in solid); apramycin, 25 µg/mL (or 50 µg/mL); hygromycin, 50 µg/mL (or 150 µg/mL); kanamycin, 25 µg/mL in liquid or solid medium. *A. mediterranei* strains were grown at 30 °C on GYM agar medium (32) for sporulation before the preparation of spore stocks, in TSB (Tryptic Soy Broth, Becton Dickinson) for DNA extraction and in MP5 (33) for rifamycin production. Conjugations between *E. coli* ET12567 harboring pUZ8002 (or pUZ8003) and *A. mediterranei* were carried out according to Kieser et al. (34) using MS medium complemented with 10 mM CaCl2 (35), instead of MgCl2. An overlay (3 mL) of soft nutrient agar (Nutrient Broth (Thermo Scientific, Dardilly, France), with 0.8% agar) containing 25 µg/mL nalidixic acid and the appropriate antibiotics for the selection of exconjugants was added after overnight incubation of the conjugation plates. The plates were incubated for 5–7 days until exconjugants clones became visible. Antibiotic concentration used for *A. mediterranei* were as follows: apramycin, 50 µg/mL; erythromycin, 75 µg/mL; hygromycin, 75 µg/mL.

### 2.2. Plasmids and DNA Manipulations

All plasmids used in this study are listed in Table 2. Details about the plasmids constructed for this study are given in Appendix A. Amplification of DNA fragments for cloning was carried out using the high-fidelity DNA polymerase Q5 (New England BioLabs: NEB, Evry, France). Taq polymerase (Qiagen, Les Ulis, France) was used for verification PCRs. All oligonucleotides used in this study were provided by IDT (Integrated DNA Technologies, Leuven, Belgium) and are listed in Table S1. Restriction and modification (ligase, kinase, etc.) enzymes were purchased from NEB or Thermo Scientific. Plasmid DNA extraction from *E. coli* was performed using NucleoSpin Plasmid kit from Macherey-Nagel (Hoerdt, France). DNA fragments were purified from agarose gels using the NucleoSpin Gel and PCR Clean-up kit from Macherey-Nagel. Genomic DNA extractions from *Amycolatopsis* and *E. coli* transformations were performed according to standard procedures [34,35].

### 2.3. Construction and Verification of A. mediterranei DSM 40773 Mutant Strains

#### 2.3.1. Marker-Free rifK Mutants

Firstly, pRIF05 was transferred to *A. mediterranei* wild-type by conjugation. Hygromycin resistant exconjugants were selected and then screened for apramycin sensitivity to obtain clones in which the *rifK* gene was replaced by the *att1Ωhyg* cassette. The genetic organization of hygromycin-resistant and apramycin-sensitive clones was checked by PCRs targeting the upstream and the downstream junctions formed by the replacement of the *rifK* gene by the *att1Ωhyg* cassette. These PCRs were performed using the following couples of primers: LS182/LS161 and LS188/LS189, respectively. For excision of the *att1Ωhyg* cassette, the plasmid pEA01 was introduced into three independent ∆*rifK::att1Ωhyg* clones. Erythromycin resistant exconjugants were selected. Hygromycin sensitive clones were then screened and the loss of plasmid pEA01 was finally obtained by successive subculturing on medium without erythromycin. The excision of the cassette and the deletion of the *rifK* gene was checked by PCR using primers hybridizing upstream and downstream of the *rifK* gene (LS172 and LS173, respectively). For each mutant clone, the presence of the scar was checked by sequencing the PCR product.

#### 2.3.2. Large-Scale Deletions

Firstly, pRIF14 was transferred to *A. mediterranei* wild-type by conjugation. Hygromycin resistant exconjugants were selected. The integration of the plasmid pRIF14 was checked by PCR using primers LS190 and LS71 targeting the downstream junction formed by the integration of pRIF14 within the region downstream of the *rif* cluster. pRIF12 was then introduced into the strain already harboring pRIF14. Apramycin and hygromycin resistant exconjugants were selected. The integration of the plasmid pRIF12 was checked by PCR using primers LS191 and LS70 targeting the upstream junction formed by the integration of pRIF12 within the region upstream of the *rif* cluster. Finally, the excision of the complete cluster was performed as previously described for the excision of the hygromycin cassette. The deletion of the complete *rif* cluster was checked by PCR using primers hybridizing upstream and downstream of the *rif* cluster (LS220 and LS221, respectively) and allowing the amplification of the scar region formed after the excision. For each mutant clone, the presence of the scar was checked by sequencing the PCR product.

### 2.4. Rifamycin Production

MP5 liquid medium (75 mL of medium in 500 mL baffled Erlenmeyer flasks) was inoculated with 5 × 10^7^ spores of the mutant or the wild-type strains. Cultures were incubated for 10 days at 30 °C under agitation (180 rpm). Cultures were centrifugated and the supernatants used for the assay of the antibacterial activity.

### 2.5. Antibacterial Activity Assays

Antibacterial activity assays were performed using two *Staphylococcus aureus* strains as indicator (one strain sensitive to rifamycin and the other resistant). Each *S. aureus* strain was grown overnight in LB at 37 °C and used to inoculate molten Antibiotic medium 5 (Becton Dickinson, Le Pont de Claix, France). Each indicator plate was loaded with 100 µL of *A.* *mediterranei* supernatants and then incubated overnight at 37 °C for growth inhibition analysis. The growth inhibition of strains by rifamycin was verified with 100 µL of MP5 containing or not rifamycin SV (5 mg/mL). The contrast between regions of normal and inhibited growth was enhanced using tetrazolium red (Sigma) as described by Pattee [38]. These assays were performed at least three times for each mutant strain.

## 3. Results

**Figure 1:**
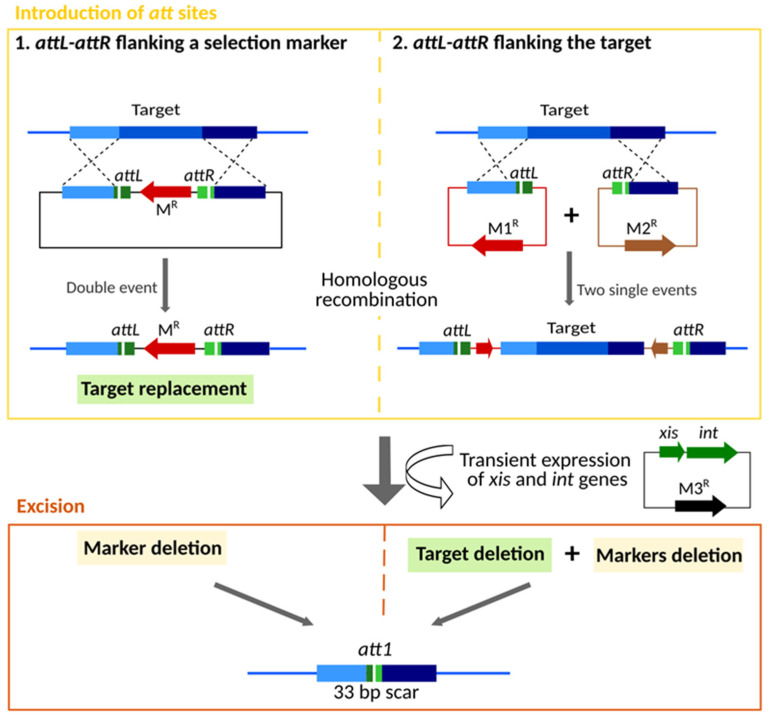
Principle of pSAM2 SSR system and examples of its application (schematic representations not to scale). The SSR system requires *cis*-acting elements (*attL* and *attR* sites) and *trans*-acting elements (*xis* and *int* genes). First, the *att* sites are introduced in the genome via homologous recombination. The *attL* and attR sites are integrated in the genome by (1) a double event of homologous recombination in which the target is replaced by an excisable cassette/marker; or (2) two single events of homologous recombination after which the target is flanked by the *att* sites. Then the *xis* and *int* genes are introduced and temporarily expressed to perform the excision of the region flanked by *attL-attR* sites.

The application of the pSAM2 SSR system requires *cis-* and *trans*-acting elements and involves two steps (Figure 1). First, the *cis* elements, the *attL* and *attR* recombination sites, are introduced in the genome via homologous recombination. Then, the *trans*-acting elements, the *xis* and *int* genes, are temporarily expressed to perform SSR and consequently the excision of the region flanked by *attL* and *attR*.

The excision step requires only the transient expression of the *int* and *xis* genes. This can be achieved by cloning these genes in a vector self-replicating in *Amycolatopsis* which is rapidly lost in the absence of a selection pressure. Considering the introduction of *attL* and *attR* sites in the genome, two approaches can be used. In the first one, the target gene(s) is replaced by an antibiotic resistance gene flanked by *attL* and *attR*. For this purpose, several excisable cassettes, previously constructed [25], are available for cloning in an *Amycolatopsis* suicide vector, in between sequences identical to the upstream and downstream regions of the target gene. This approach was originally designed to inactivate a single gene by *in-frame* deletion for functional analysis [39]. It can also be used to delete a few adjacent genes. Even if we used this approach to delete small (<30 kb) biosynthetic gene clusters (BGCs), such as the bicyclomycin BGC [40] and the congocindine BGC [41], the efficiency of the target replacement (double event of recombination) by homologous recombination decreases with the size of the target region. In this context, for large-scale deletions we propose here a second approach in which *attL* and *attR* sites are successively introduced at the borders of the target region by homologous recombination (two single events). For this propose *attL* and *attR* sites must be cloned in two compatible non-replicative vectors, within the identical sequences required for homologous recombination between the vector and the host genome.

Therefore, in this work we constructed a set of three vectors, one self-replicative in *Amycolatopsis* for transient expression of the *xis* and *int* genes, and two suicide vectors for the integration of the recombination sites *attL* and *attR* on each side of target region to be deleted. The application of this tools in *Amycolatopsis* has then evaluated for single gene or large-scale deletions in *A. mediterranei* DSM 40773.

### 3.1. Design of Genetic Tools for pSAM2 SSR System Application

**Figure 2:**
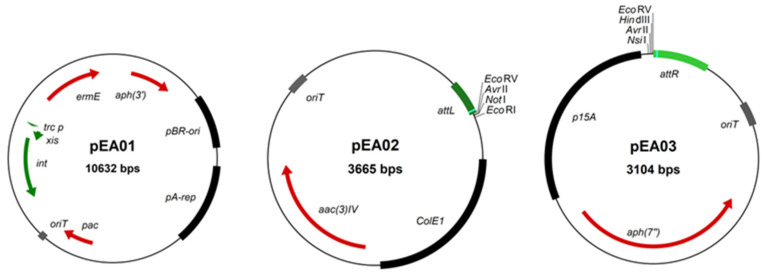
Maps of the plasmids constructed in this study. All three plasmids are replicative in *E. coli.* pEA01 can replicate in *Amycolatopsis* while pEA02 and pEA03 are suicide vectors in *Amycolatopsis. aph(3′)*: kanamycin resistance gene; pBR-ori: pBR322 origin of replication; pA-rep: short replicon region of pA387; *pac*: puromycin resistance gene; *oriT*: origin of transfer; *int* and *xis*, integrase and excisionase gene of pSAM2; *trc*_p_*:* trc promoter for the expression of *xis* and *int* genes; *ermE*: erythromycin resistance gene; *attL:* left attachment site; ColE1: ColE1 origin of replication; *aac(3)IV*: apramycin resistance gene; *aph(7″*): hygromycin resistance gene; p15A: p15A origin of replication; *attR*: right attachment site.

For transient expression of the *int* and *xis* genes in *Amycolatopsis*, we constructed a plasmid relying on the short form of the pA387 replicon contained in pRL60 [29] for replication in *Amycolatopsis*. If fact, pRL60 has been reported to be unstable in the absence of a selection pressure [42]. The presence of the origin of transfer *oriT* allows the interspecific transfer of the plasmid from *E. coli* strain to *Amycolatopsis* species by conjugation, as widely performed in actinobacteria. In our plasmid, three antibiotic resistance genes are present, conferring kanamycin, erythromycin or puromycin resistance, allowing its selection in various *Amycolatopsis* genetic backgrounds. The kanamycin marker is used for selection in *E. coli* strains. The *xis* and *int* genes are placed under the control of the promoter *trc*_p_, a promoter that efficiently expresses these genes in *Streptomyces* spp [25]. The resulting plasmid possessing all these features was called pEA01 (Figure 2). This plasmid is available from the Addgene repository (ID 170766).

The study of the vector replication, selection and stability was performed in *A. mediterranei* DSM 40773. After conjugative transfer of pEA01 to *A. mediterranei* DSM 40773, exconjugants resistant to erythromycin and puromycin were obtained. Of note, the kanamycin marker was not useful for this strain since *A. mediterranei* DSM 40773 is spontaneously resistant to kanamycin. The instability of pEA01 was assessed in absence of antibiotic selection: After 3 days of growth in media without antibiotic, 99.5% of clones (995 of 1000 analyzed) were sensitive to erythromycin indicating the loss of pEA01 vector. This confirmed that the short pA387 replicon from pRL60 is suitable to generate replicative but unstable vectors in *Amycolatopsis,* as required for transient expression of genes.

For successive introduction of *attL* and *attR* sites at the borders of the target region by homologous recombination, we designed two non-replicative vectors that share minimal sequence identity to avoid recombination between them. As both plasmids are conjugative, they nevertheless share an identical region corresponding to the *oriT* sequence (≈500 bp). This is the only long stretch of identity as we used different replicons (ColE1 and p15A) for replication in *E. coli* and different antibiotic resistance genes (apramycin and hygromycin resistance). The two vectors pEA02 and pEA03, contain the *attL* and *attR* sites, respectively (Figure 2). They both harbor a multiple cloning site for the easy cloning of the sequences required for homologous recombination. These vectors are suitable for introduction of *attL* and *attR* sites at any target borders by a single event of homologous recombination. Detailed instructions to clone homologous regions into these vectors are provided in Figure S1. pEA02 and pEA03 are available from Addgene (ID 172193 and ID 172194, respectively).

The set of three vectors and the efficiency of each approach were thereafter evaluated for single gene or large-scale deletions in *Amycolatopsis*. We used as target the rifamycin biosynthetic gene cluster of *A. mediterranei* DSM 40773.

### 3.2. Cassette Excision

**Figure 3:**
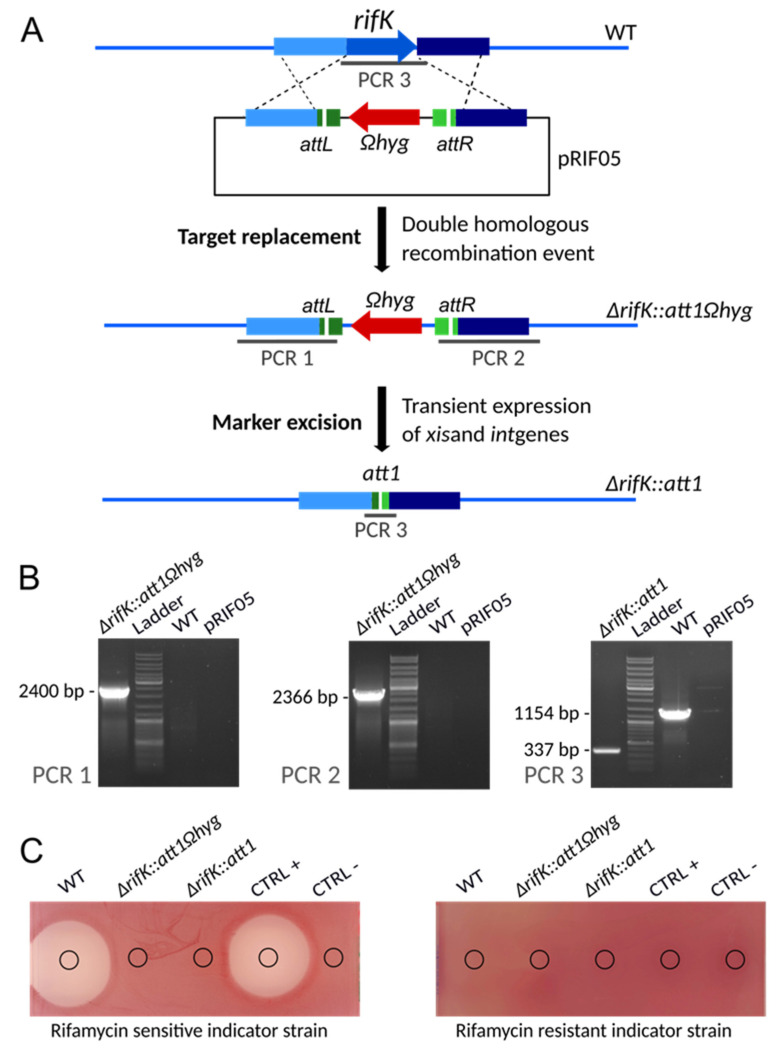
Construction of an unmarked *rifK* deletion mutant using an excisable cassette. (**A**) Schematic representation (not to scale) of the successive steps. First the *rifK* gene is replaced by the *att1Ωhyg* cassette via a double homologous recombination event. Then the *att1Ωhyg* cassette is excised following the introduction of pEA01, resulting in an unmarked deletion mutant containing a 33-bp scar (*att1*). (**B**) PCR verification of the strains at different stages of the construction. Verifications are based on three PCRs. The extent of the expected amplicons for each of the PCR is indicated in panel A. The amplicons sizes are indicated on the left side of the pictures. Lanes: ∆*rifK::att1Ωhyg*- genomic DNA from one of the *A. mediterranei* DSM43770 ∆*rifK::att1Ωhyg* clones, representative of all analyzed clones; Ladder-GeneRuler DNA Ladder (SM0331 Thermo Scientific); ∆*rifK::att1*-genomic DNA from one of the *A. mediterranei* DSM43770 ∆*rifK::att1* clones, representative of all analyzed clones WT-genomic DNA from *A. mediterranei* DSM43770 wild-type strain; pRIF05-Plasmid pRIF05. (**C**) Bioassays. The antibacterial activity of the culture supernatants from the wild-type and the mutant strains of *A. mediterranei* DSM43770 was assayed against *S. aureus* HG003 (sensitive to rifamycin) and its *rpoB*_H481Y_ mutant derivative (resistant to rifamycin). MP5 with or without 5 µg/mL of rifamycin SV was used as positive or negative control, respectively.

The strategy employed for the obtention of marker-free *rifK* mutants is summarized in Figure 3A. First *rifK* was replaced by an excisable hygromycin resistance cassette (*att1Ωhyg*) following double homologous recombination events between the non-replicative plasmid pRIF05 and the host genome. For that, pRIF05 containing the excisable cassette between the upstream and the downstream homologous regions was introduced into the wild-type strain by conjugation. Hygromycin resistant exconjugants were selected and then tested for sensitivity/resistance to apramycin. Of the 300 hygromycin resistant clones tested, 20 were sensitive to apramycin, indicating that double homologous recombination events occur with a frequency (about 7%) that makes them relatively easy to obtain. These hygromycin-resistant and apramycin-sensitive clones were then checked by PCR on genomic DNA. The verification is based on the amplification of the junctions formed between the upstream or the downstream region of *rifK* and the *att1Ωhyg* cassette. As expected only the clone of interest allowed the amplification of DNA fragments with the expected size (PCR 1 and PCR 2 in Figure 3B). The results are shown for one clone, representative of the 20 clones analyzed. Thus, in all the 20 clones, the expected recombination events had occurred. The resulting clones were called *A. mediterranei* DSM 40773 ∆*rifK::att1Ωhyg*, and three of them were used in further steps and for phenotypic analysis.

In the second step, the excision of the *att1Ωhyg* cassette was performed by introducing the plasmid pEA01 in three independent ∆*rifK::att1Ωhyg* clones. The erythromycin resistant exconjugants obtained were then screened for resistance/sensitivity to hygromycin to check the loss of the hygromycin cassette. Over the 150 (50 from each parental clone) clones tested 147 were sensitive to hygromycin indicating that the excision occurs with a frequency of 98%. In this case, 18 of these hygromycin sensitive clones (6 from each parental clone) were genetically verified by PCR, targeting the scar region formed by the excision of *Ωhyg* cassette. As expected, a smaller fragment was amplified with genomic DNA of the sensitive hygromycin clones when compared to the 1154 bp fragment obtained with the wild-type genomic DNA. The results obtained for PCR 3 are also shown in Figure 3B for one clone, representative of the 18 clones tested. The sequence analysis of the 337 bp amplicon, confirmed the excision of the *Ωhyg* cassette and the formation of a 33 bp scar, corresponding to the *att1* sequence [25], as expected. Sanger sequencing results obtained for three independent clones are provided in Supplemental File S1. Moreover, these clones became sensitive to erythromycin, indicating that pEA01 has been lost during cultivation in the absence of erythromycin. The resulting clones were called *A. mediterranei* DSM 40773 ∆*rifK::att1*, and three of them (one from each parental clone) were used for phenotypic analyses.

Phenotypic analysis, presented in Figure 3C, was performed using a bacterial growth inhibition assay. The results shown that ∆*rifK::att1Ωhyg* and ∆*rifK::att1* strains did not produce rifamycin contrarily to the wild-type strain, confirming that this gene is essential for antibiotic production.

### 3.3. Region Excision

**Figure 4:**
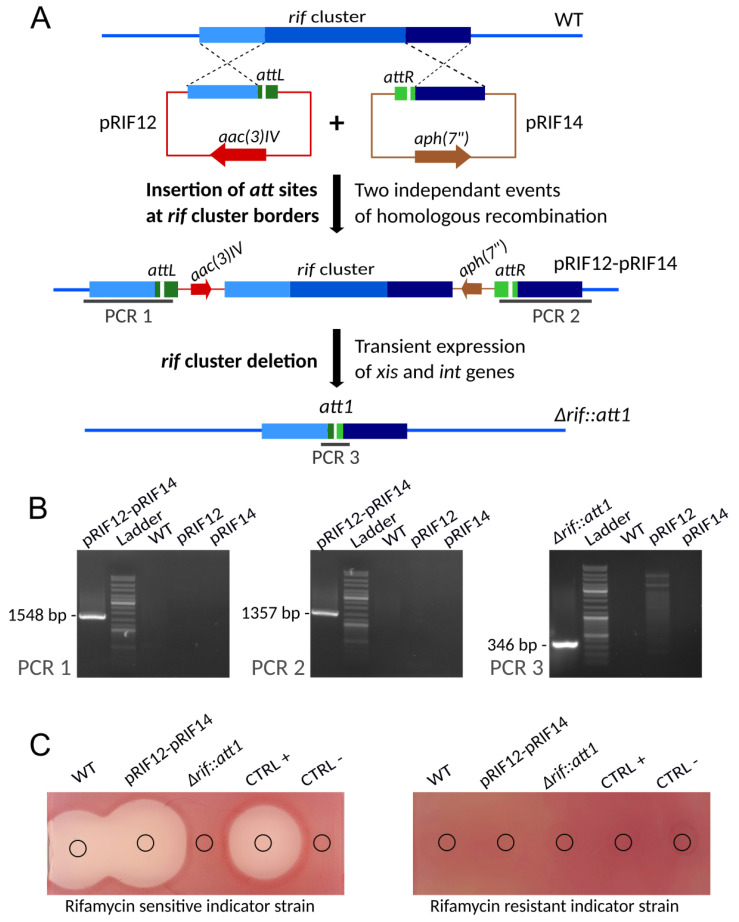
Construction of an unmarked *rif* cluster deletion mutant. (**A**) Schematic representation (not to scale) of the successive steps. First the *attL* and *attR* sequences are successively integrated upstream and downstream of the *rif* cluster via two single homologous recombination events. Then the complete region between the *attL* and *attR* sites is excised following the introduction of pEA01, resulting in an unmarked *rif* cluster deletion mutant containing a 33-bp scar (*att1*). (**B**) PCR verification of the strains at different stages of the construction. Verifications are based on three PCRs. The extent of the expected amplicons for each of the PCR is indicated in panel A. Lanes: pRIF12-pRIF14-genomic DNA of *A. mediterranei* DSM43770 harboring pRIF12 and pRIF14, representative of all analyzed exconjugants; Ladder-GeneRuler DNA Ladder (SM0331 Thermo Scientific); WT-genomic DNA of *A. mediterranei* DSM43770 wild-type strain; pRIF12-Plasmid pRIF12; pRIF14-Plasmid pRIF14; ∆*rif::att1*-genomic DNA of *A. mediterranei* with a deletion of the *rif* cluster. (**C**) Bioassays. The antibacterial activity of culture supernatants from the wild-type strain *A. mediterranei* DSM43770, the strain harbouring pRIF12 and pRIF14, and the mutant strain ∆*rif::att1* was assayed against *S. aureus* HG003 (sensitive to rifamycin) and its *rpoB*_H481Y_ derivative (resistant to rifamycin). The slight difference in inhibition zone size observed for WT and pRIF12-pRIF14 strains is due to the fluctuation of rifamycin production between different flasks. MP5 with or without 5 µg/mL of rifamycin SV was used as positive or negative control, respectively.

The complete rifamycin gene cluster was targeted to demonstrate the use of the pSAM2 SSR system to generate marker-free large-scale deletions. The rifamycin biosynthetic gene cluster comprises 42 genes covering a region of about 90 kb [43]. The strategy employed to delete this cluster is shown in Figure 4A. First the *attL* and *attR* sequences were integrated at the cluster’ extremities via homologous recombination. In a second step, the excision of the region flanked by *attL-attR* sites and encompassing the rifamycin gene cluster was performed using the pEA01 vector.

For this purpose, fragment identical to the region upstream and downstream of the rifamycin cluster were cloned into pEA02 and pEA03, yielding pRIF12 and pRIF14, respectively. These plasmids were then transferred into the wild-type strain by successive conjugations. After conjugative transfer of pRIF14 into the wild-type strain, clones resistant to hygromycin were selected. These clones were then verified by PCR on genomic DNA to confirm the integration of pRIF14 at the downstream extremity of rifamycin cluster. In a second step, pRIF12 was transferred into the strain already carrying the pRIF14, and clones resistant to apramycin and hygromycin were selected. Then, as previously described for strains carrying the pRIF14, these clones were verified by PCR. The results obtained confirmed the integration of pRIF12 and pRIF14 at the expected chromosomal sites (upstream and downstream to the rifamycin gene cluster) as presented in Figure 4B for PCR 1 and PCR 2. The resulting clones were called *A. mediterranei* DSM 40773-pRIF12-pRIF14 and were used in further steps and for phenotypic analysis.

The excision of the rifamycin gene cluster was performed in two independent *A. mediterranei* DSM 40773-pRIF12-pRIF14 clones, using the plasmid pEA01. The erythromycin resistant clones obtained after conjugation of pEA01 were then screened for resistance/sensitivity to hygromycin and/or apramycin to check the loss of hygromycin and/or apramycin cassette. Over the 100 tested exconjugants (50 from each parental clone) that received pEA01 vector, 95 were sensitive to both antibiotics indicating that the excision occurs with a frequency of 95%. In this case, 12 of these sensitive clones (6 from each parental clone) were verified by PCR, targeting the scar region formed after excision (PCR 3 in Figure 4B). As expected, a DNA fragment of 346 bp, containing the *att1* sequence [25], was amplified for all the 12 clones tested. Sanger sequencing results obtained for three independent clones are provided in Supplemental File S2. Moreover, these clones became sensitive to erythromycin, indicating that pEA01 has been lost during cultivation in the absence of erythromycin. The resulting clones were called *A. mediterranei* DSM 40773 ∆*rif::att1*, and three of them were used for phenotypic analyses.

As previously described for *rifK* mutants, bacterial growth inhibition assay results confirmed that the ∆*rif::att1* strains did not produce rifamycin, as shown in Figure 4C. The integration of pRIF12 and pRIF14 at the border of the cluster does not affect the production of rifamycin.

This experiment validates the functionality of the set of vectors developed in this work and the application of the pSAM2 SSR system to generate large-scale deletions in *A. mediterranei* DSM 40773.

## 4. Discussion

The genetic tools that we constructed allow the application of the pSAM2 SSR system in *A. mediterranei* to generate marker-free small or large deletions. The transient expression of the *xis* and *int* genes was achieved by using an unstable replicative vector which is very rapidly lost in the absence of a selection pressure. No additional step of growth in the absence of selection was required to lose the excision plasmid pEA01. It should also be noted that with the delivery suicide vectors that we used (pOJ260, pEA02, pEA03), we did not observed integration by illegitimate recombination as was sometimes the case in *Amycolatopsis* with other vectors [7,8]. The SSR between *attL* and *attR* leading to the excision of the region flanked by these sites is highly efficient (>95%). The efficiency is roughly the same whether these sites are close (98% efficiency with excisable cassettes) or quite distant (e.g., 95% efficiency for sites separated by 90 kb). These tools allow the use of resistance marker for steps where a selection is helpful and the subsequent removal of these markers by SSR to obtain marker-less deletion mutant strains. This allows the recycling of resistance markers for successive rounds of genetic engineering, an advantage for a genus for which the number of usable resistance markers is limited. Moreover, the absence of heterologous antibiotic resistance genes might be important for biotechnological applications. In addition, unmarked *in-frame* gene deletion prevents polar effects on the expression of downstream genes [25].

The application of pSAM2 SSR system to generate unmarked deletion mutants requires the introduction of several vectors into *Amycolatopsis*: (1) the introduction of either a suicide vector for gene replacement by a selection marker or two suicide vectors for the introduction of *attL* and *attR* at the borders of the target region; (2) the introduction of an unstable self-replicative vector for excision. Thus, this process together with the recently reported CRISPR-Cas12a based method [13], which requires the successive introduction of two constructions, might seem more time-consuming than the ones using double homologous recombination techniques associated to a chromogenic screen [7] or a counterselectable marker [8], which require the introduction of a single vector. In these systems, the integration of a suicide vector by simple crossing over is selected using a resistance marker and then the loss of the vector, after a second event of homologous recombination, can be screen by the colour of the colonies or selected using the counterselectable marker. However, obtaining clones in which a second event of homologous recombination had occurred may require several rounds of sporulation in non-selective media [7], chromogenic screening can be hampered by the natural pigmentation of the colonies in some strains [8] and the use of counterselectable markers often implies to use a mutant strain [8]. With the approach described in this work, the steps based on homologous recombination are always associated with the possibility of selecting or screening the desired events by antibiotic resistance, a simple and robust procedure. The excision step, using the pSAM2 SSR system, results in a very high a very high (>95%) excision frequency, so that selection for the desired mutant is not necessary. After excision, apart a small scar of 33 bp, no exogenous DNA is left in the final strain. The CRISPR-Cas12a genome editing system recently described in *A. mediterranei* [13] has the advantage of not leaving any scar. However, the CRISPR-Cas12a edited *A. mediterranei* strains still carries the *cas12a* and the hygromycin resistance genes integrated in its chromosome [13]. In addition to *A. mediterranei*, we have been using these tools with success in two other *Amycolatopsis* species, *Amycolatopsis tucumanensis* [44] and *Amycolatopsis* sp. AA4 [45]. Moreover, the pSAM2 SSR system should be functional in many *Amycolatopsis* strains as no proteins other than Xis and Int are required for SSR, these proteins are expressed from a orthogonal promoter (trc_p_) which should not be affected by endogenous regulations from the host, and the pSAM2 SSR system, expressed from this same promoter, has previously been shown to be functional in several *Streptomyces* species (e.g., *Streptomyces coelicolor* A3(2) [46], *Streptomyces lividans* [47], *Streptomyces venezuelae* [48]) and in *E. coli* [23]. Therefore, the genetic tools developed here enrich the toolbox available for genome engineering in *Amycolatopsis* spp. They offer a simple, robust, and efficient alternative to other systems using chromogenic screening [7], counterselectable markers [8] or based on CRISPR-Cas technology [13] and should be helpful for the exploration and exploitation of *Amycolatopsis* spp. biotechnological potential. Moreover, it should be noted that the plasmids pEA02 and pEA03 might be used in *Streptomyces* spp. in combination with the *Streptomyces* replicative vectors expressing Xis and Int (pOSV236 [36], pOSV507 or pOSV508 [25]) to obtain large-scale deletions.

## Figures and Tables

**Table 1:** Strains used in this study.

Strain	Description	Reference or Source
*E. coli* DH5α	General cloning strain	Promega
*E. coli* ET12567/pUZ8002	Host strain for conjugation from *E. coli* to *Amycolatopsis*	[30,31]
*E. coli* ET12567/pUZ8003	Host strain for conjugation from *E. coli* to *Amycolatopsis* (pUZ8003 is a modified pUZ8002 with *aph(3′)* gene replaced by *bla*)	[32]
*A. mediterranei* DSM 40773	Wild-type (WT) strain	DSMZ
DSM 40773 ∆*rifK::att1Ωhyg*	*A. mediterranei rifK* deletion mutant with replacement of the *rifK* gene by the *att1Ωhyg* hygromycin resistance cassette	This study
DSM 40773 ∆*rifK::att1*	Unmarked *A. mediterranei rifK* deletion mutant	This study
DSM 40773-pRIF14	*A. mediterranei* containing pRIF14	This study
DSM 40773-pRIF12-pRIF14	*A. mediterranei* containing both plasmids pRIF14 and pRIF12	This study
DSM 40773 ∆*rif::att1*	*A. mediterranei* with a deletion of the complete *rif* cluster	This study
*S. aureus* HG003	Rifamycin sensitive strain used as indicator in bioassay analysis	[33]
*S. aureus* HG003 *rpoB* (H418Y)	Rifamycin resistant strain used as indicator in bioassay analysis, *rpoB* mutant of *S. aureus* HG003	Marick Esberard and Philippe Bouloc unpublished

**Table 2:** Plasmids used in this study.

Plasmid	Description ^a^	Reference or Source
pRL60	*E.coli-Amycolatopsis* shuttle plasmid, Ery ^R^, Kan ^R^, amy^+^, *oriT*	[29]
pOSV236	*E. coli-Streptomyces* shuttle plasmid expressing the Xis and Int proteins for site-specific excision of excisable cassettes, Amp ^R^, Pur ^R^, *oriT*	[36]
pOSV504	Source of the excisable hygromycin cassette (*att1Ωhyg*), Amp ^R^, Hyg ^R^	[25]
pOJ260	*E.coli-Amycolatopsis* shuttle plasmid, suicide vector in *Amycolatopsis*, Apr ^R^, *oriT*	[37]
pOSV805	*E.coli-Amycolatopsis* shuttle plasmid, integrative in *Amycolatopsis* using the ϕBT1 SSR system, Hyg ^R^, *oriT*	[32]
pCR-blunt	*E. coli* cloning vector, Kan ^R^	Invitrogen
pT-atts	pUC derivative containing the *attL* and *attR* minimal sites from pSAM2, Amp ^R^, Constructed by Twist Biosciences	This study
pRL60∆*amy*	*E.coli-Amycolatopsis* shuttle plasmid, derivative of pRL60, amy^−^, Ery ^R^, Kan ^R^, oriT	This study
pEA01	*E.coli-Amycolatopsis* shuttle plasmid, pRL60∆*amy* derivative containing the *xis* and *int* genes from pSAM2 under the control of *trc*_p_, the *pac* gene and *oriT*, Ery ^R^, Kan ^R^, Pur ^R^, *oriT*	This study
pEA02	*E.coli-Amycolatopsis* shuttle plasmid, suicide vector in *Amycolatopsis*; pOJ260 derivative containing the minimal *attL* sequence and a multi-cloning site, Apr ^R^, *oriT*	This study
pEA03	*E.coli-Amycolatopsis* shuttle plasmid, suicide vector in Amycolatopsis; pOSV805 derivative containing the minimal *attR* sequence and a multi-cloning site, Hyg ^R^, *oriT*	This study
pRIF01	pCR-blunt derivative containing the downstream homologous region of *rifK*, KanR	This study
pRIF02	pCR-blunt derivative containing the upstream homologous region of *rifK*, KanR	This study
pRIF05	pOJ260 derivative containing the excisable hygromycin cassette (*att1Ωhyg*) flanked by the upstream and downstream homologous region of *rifK*, for replacement of *rifK*, Apr ^R^, *oriT*	This study
pRIF09	pCR-blunt derivative containing the downstream homologous region of the *rif* cluster, Kan ^R^	This study
pRIF10	pCR-blunt derivative containing the upstream homologous region of the *rif* cluster, Kan ^R^	This study
pRIF12	*E.coli-Amycolatopsis* shuttle plasmid, suicide plasmid in *Amycolatopsis*; pEA02 derivative containing the upstream region of the *rif* cluster, *attL*, Apr ^R^, *oriT*	This study
pRIF14	*E.coli-Amycolatopsis* shuttle plasmid, suicide plasmid in *Amycolatopsis*; pEA03 derivative containing the downstream region of the *rif* cluster, *attR*, Hyg ^R^, *oriT*	This study

^a^ Abbreviations: amy—α-amylase; ^R^—Resistance, Ery—Erythromycin, Kan—Kanamycin, Amp—Ampicillin, Pur—Puromycin, Apr—Apramycin, Hyg—Hygromycin.

## Data Availability

The genetic tools described here are available from Addgene (www.addgene.org, accessed on 15 March 2022), where information (e.g., maps and sequences) can be found.

## Supplementary Materials

The following supporting information can be downloaded at https://www.mdpi.com/article/10.3390/microorganisms10040828/s1, Figure S1: Large-scale marker-free deletions using the pSAM2 SSR system. Table S1: Oligonucleotides used in this study. File S1: Sequence data of marker-free *rifK* mutants. File S2: Sequence data of marker-free *rif* cluster mutants.

## Appendix A

**Plasmid Construction**

(i) Construction of the plasmid pEA01 expressing Xis and Int

pRL60 was digested by *Eco*RI and DNA fragments of 6 kb (*aph(3′)* gene, pBR*-*origin and pA-rep) and 1,8 kb (*ermE* gene) were then purified and ligated, yielding pRL60∆*amy.* The DNA fragment containing the *xis* and *int* genes under control of *trc*_p_, the puromycin resistance gene *pac* and the origin of transfert *oriT* was isolated from pOSV236 by digestion with *Not*I and *Ssp*I. This 3 kb fragment was then blunt-ended using Klenow enzyme and cloned into pRL60∆*amy* linearized by *Eco*RV and dephosphorylated, yielding pEA01. The construction was verified by restriction enzyme digestion and sequencing.

(ii) Construction of the plasmids pEA02 and pEA03 carrying *attL* and *attR*

A DNA fragment containing the *attL* and *attR* sites surrounded by desired restriction sites was chemically synthesized and cloned into a high copy number plasmid by Twist Bioscience, yielding pT-atts. A *Bam*HI/*Eco*RI fragment containing the *attL* sequence flanked by the *Eco*RV, *Avr*II and *Not*I restriction sites was isolated from pT-atts and cloned into pOJ260 linearized by *Bam*HI and *Eco*RI, yielding pEA02. A *Pst*I*/Nsi*I fragment containing the *attR* sequence flanked by the *Eco*RV, *Hin*dIII and *Avr*II restriction sites was isolated from pT-atts and ligated to a 2.8 kb fragment obtained by digestion of pOSV805 by *Pst*I and *Nsi*I, yielding pEA03. It should be noted that the *int* gene from ϕBT1, present in pOSV805, is no longer present in pEA03. Thus, pEA03 is a suicide vector in *Amycolatopsis*, as pEA02. Both plasmids were verified by restriction analyzes and partial sequencing.

(iii) Construction of pRIF05 for the inactivation of *rifK*

The regions upstream and downstream (≈2 kb each) of *rifK* were amplified by PCR using the following couple of primers: LS151/LS152 and LS153/LS154, respectively. Each PCR product was cloned into pCR-blunt, yielding pRIF02 and pRIF01. Effective cloning of the fragments in pCR-blunt was controlled by restriction analysis and the integrity of the insert was verified by sequencing. The downstream region was then isolated from pRIF01 as a *Hin*dIII/*Pvu*II fragment, while the upstream region was isolated from pRIF02 as a *Bam*HI/*Pvu*II fragment. The *att1Ωhyg* cassette was isolated from pOSV504 by *Eco*RV. The three DNA fragments were then cloned together into pOJ260 digested by *Bam*HI and *Hin*dIII, yielding pRIF05.

(iv) Construction of pRIF12 and pRIF14 for deletion of the rifamycin gene cluster

The regions upstream (1.2 kb) and downstream (1 kb) of the rifamycin (*rif*) biosynthetic gene cluster were amplified using the pairs of primers: LS155/LS156 and LS157/LS158, respectively. Each PCR product was cloned in pCR-blunt, yielding pRIF10 and pRIF09. Effective cloning of the fragments in pCR-blunt was controlled by restriction analysis and the integrity of the insert was verified by sequencing. The *Eco*RV/*Not*I fragment of pRIF10 containing the upstream region of the *rif* cluster was then cloned into pEA02 digested by the same enzymes yielding pRIF12. In the same way, to construct pRIF14, the downstream region of the *rif* cluster was isolated from pRIF09 as a *Nsi*I/*Eco*RV fragment and was then cloned into pEA03 digested by the same enzymes.
