# Supplementary material for: Marker-Free Genome Engineering in Amycolatopsis Using the pSAM2 Site-Specific Recombination System
Source: Microorganisms. 2022 Apr 16;10(4):828. doi: 10.3390/microorganisms10040828 (PMC9033027; doi:10.3390/microorganisms10040828)
Supplement: Supplementary file 1 [file microorganisms-10-00828-s001.zip › Figure S1. Large-scale marker-free deletions using the pSAM2 SSR system.pdf]

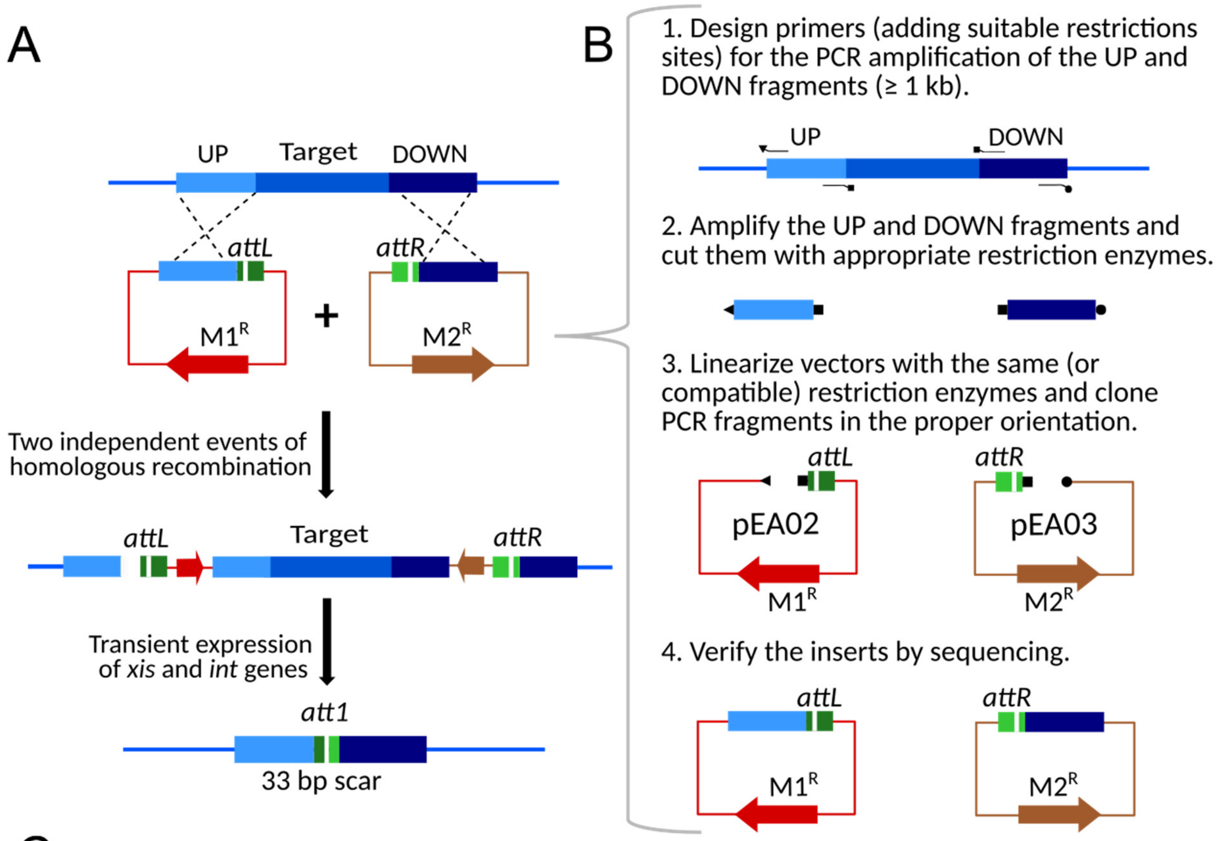

C

1. Transform *E. coli* donor strain (here ET12567/pUZ8002) with the constructed suicide vectors derived from pEA02 and pEA03.
2. Perform conjugation between *E. coli* ET12567/pUZ8002 transformed with one of the suicide vectors (e.g. pEA02 derivative) and *Amycolatopsis* strain.
3. Select apramycin resistant clones and check the correct integration by PCR.
4. Grow *Amycolatopsis/attL* clones until sporulation to prepare spore stocks.
5. Perform conjugation between *E. coli* ET12567/pUZ8002 transformed with the other suicide vector (e.g. pEA03 derivative) and *Amycolatopsis/attL* strain.
6. Select hygromycin resistant clones and check the correct integration by PCR.
7. Grow *Amycolatopsis/attL-attR* clones until sporulation to prepare spore stocks.

Of note, instead of two successive conjugations, a single conjugation using two donor strains, each one containing one of the two suicide vectors, might be performed. In this case, a selection with both antibiotics is required in step 3 and the correct integration of both vectors should be verified, before going directly to step 7.

8. Transform *E. coli* donor strain (here ET12567/pUZ8003) with pEA01 and perform conjugation with *Amycolatopsis/attL-attR* clones.
9. Select erythromycin (or kanamycin or puromycin) resistant clones and screen their sensitivity to apramycin and hygromycin.
10. Check the genetic organization on the excised (apramycin and hygromycin sensitive) clones by PCR and sequencing.
11. Grow the verified *Amycolatopsis/Δtarget::att1* clones until sporulation to prepare spore stocks.

Figure S1. Large-scale marker-free deletions using the pSAM2 SSR system. **A)** Schematic representation of the successive steps for large region excision (not to scale). First, the minimal *attL* and *attR* sites are successively introduced at the borders of the target region by homologous recombination. *att* sites are represented by a green rectangle in which the white line represents the crossover sequence (TCGGG) where SSR takes place. For proper excision of the large region (target region, vectors and markers used to introduce the *att* sites), these *att* sites should be external to this large region. Then, the deletion is performed by the expression of the *xis* and *int* genes. The intramolecular SSR between minimal *attL* and *attR* sites leads to the formation of the *att1* site. **B)** Main steps to clone the homologous fragments (identical fragments to the upstream (UP) and the downstream (DOWN) regions) into pEA02 and pEA03 for the correct integration of *att* sites at the target borders. ◄ Represents *Avr*II, *Not*I, *Eco*RI (or compatible) restriction sites; ■ Represents *Eco*RV (or any blunt end) restriction site or no restriction site (for blunt end PCR products) ● Represents *Hin*dIII, *Avr*II, *Nsi*I (or compatible) restriction sites. **C)** Stepwise overview of large-scale deletion protocol.
