# Supplementary material for: Marker-Free Genome Engineering in Amycolatopsis Using the pSAM2 Site-Specific Recombination System
Source: Microorganisms. 2022 Apr 16;10(4):828. doi: 10.3390/microorganisms10040828 (PMC9033027; doi:10.3390/microorganisms10040828)

File S1: Sequence data of marker-free *rifK* mutants

Nucleotide sequence and chromatograms obtained after Sanger sequencing of three DSM 40773 Δ*rifK::att1* independent clones are provided hereafter. The 33 bp scar expected to be formed after excision is highlighted in green. The high quality of the sequence shown in the chromatograms confirmed the homogeneity of the samples.

>mutΔrifKatt1_cl1-LS172_G01.ab1

TCTTCGGCCTGGGTTGACGAACCGGCCACTACCAAGCCGAGGGATGTAAGCC
GCCCCGGAGCCGATCTAGCCTGGTGGGGACGCTGATTCCGAGGCATTCCGGA
CACGGCACGGGCTATTTCTGCAATTCGAACCCCAGCGGAGATTCGGAGAACC
ATGAACGCGCGAAAGGCACCGGAATTCCCAGATCGCGCGCTTCGTTCGGGAC
GAAGAGGTAGATCTGAGCCGGAGCTGCACGCGACCGCCGAAATCATCGCCG
ACGCCGTGGCGCGGGCATGAGTGATCCTCGA

>mutΔrifKatt1_cl2-LS172_I01.ab1

CGTCTCGTGATTTCTTCGGCCTGGGTTGACGAACCGGCCACTACCAAGCCGA
GGGATGTAAGCCGCTTCGGAGCCGATCTAGCCTGGTGGGGACGCTGATTCCG
AGGCATTCCGGACACGGCACGGGCTATTTCTGCAATTCGAACCCCAGCGGAG
ATTCGGAGAACCATGAACGCGCGAAAGGCACCGGAATTCCCAGATCGCGCG
CTTCGTTCGGGACGAAGAGGTAGATCTGAGCCGGAGCTGCACGCGACCGCC
GAAATCATCGCCGACGCCGTGGCGCGGGCTGAGGGATCCTCGA

> mutΔrifKatt1_cl3-LS172_A01.ab1

CAAGTCATGGATCTATCGCGATTTCTTCGGCCTGGGTTGACGAACCGGCCCTA
CCAGCCGAGGGATGTAAGCCGCTCGGAGCCGATCTAGCCTGGTGGGGACGC
TGATTCCGAGGCATTCCGGCAGGCACGGGCTATTTCTGCAATTCGAACCCCA
GCGGAGATTCGGAGAACCATGAACGCGCGAAAGGCACCGGAATTCCCAGAT
CGCGCGCTTCGTTCGGGACGAAGAGGTAGATCTGAGCCGGAGCTGCACGCG
ACCGCCGAAATCATCGCCGACGCCGTGGCGCGGGCTGGGGGATCCTCGAAA

File: mutΔrifKatt1_cl1-LS172.ab1
Signal G:5337 A:6281 T:4544 C:9796
Sample: mutΔrifKatt1_cl1-LS172
290 bases in 3710 scans

Run Ended: Jan 7, 2021, 1:21:10
Comment: 2389291-G01-EmmanuelleDarbon-50-463277508-ED1
Lane: 9 Base spacing: 14.79

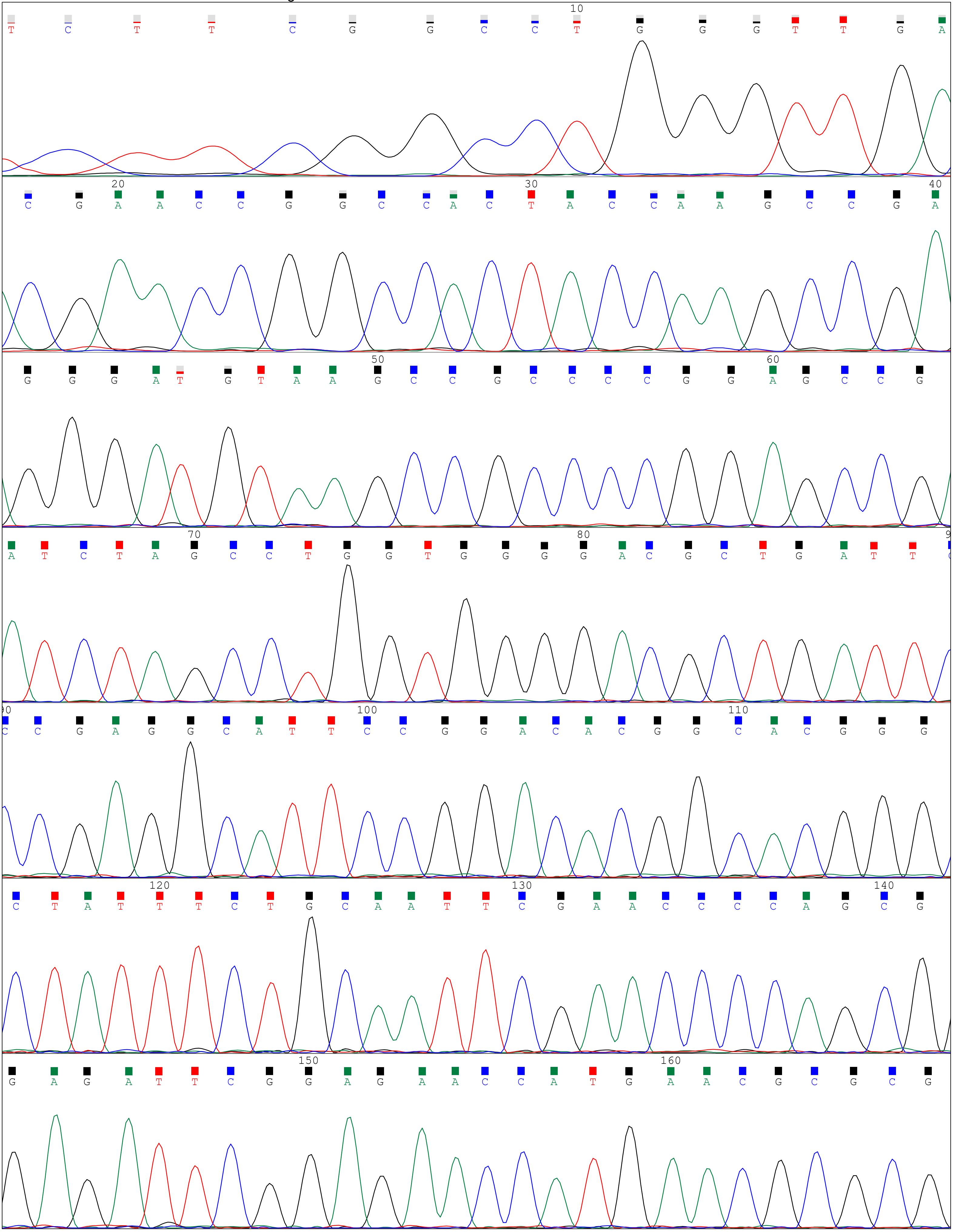

File: mut∆rifKatt1_cl1-LS172.ab1
Signal G:5337 A:6281 T:4544 C:9796
Sample: mut∆rifKatt1_cl1-LS172
290 bases in 3710 scans

Run Ended: Jan 7, 2021, 1:21:10
Comment: 2389291-G01-EmmanuelleDarbon-50-463277508-ED1
Lane: 9 Base spacing: 14.79

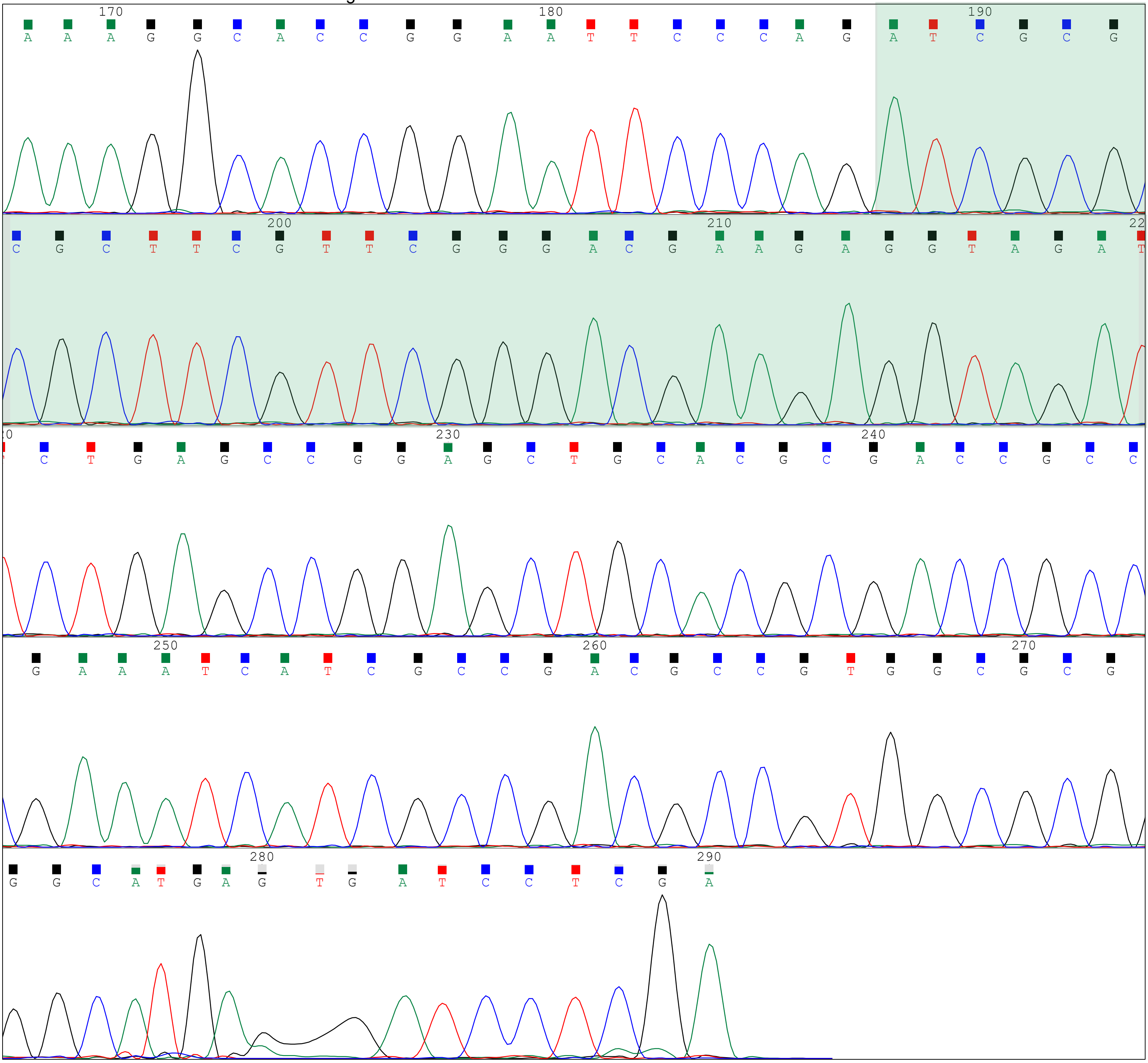

File: mutΔrifKatt1_cl2-LS172.ab1
Signal G:9720 A:11140 T:8657 C:16011
Sample: mutΔrifKatt1_cl2-LS172
301 bases in 3841 scans

Run Ended: Jan 7, 2021, 1:21:10
Comment: 2389291-I01-EmmanuelleDarbon-50-463277508-ED2
Lane: 7 Base spacing: 14.68

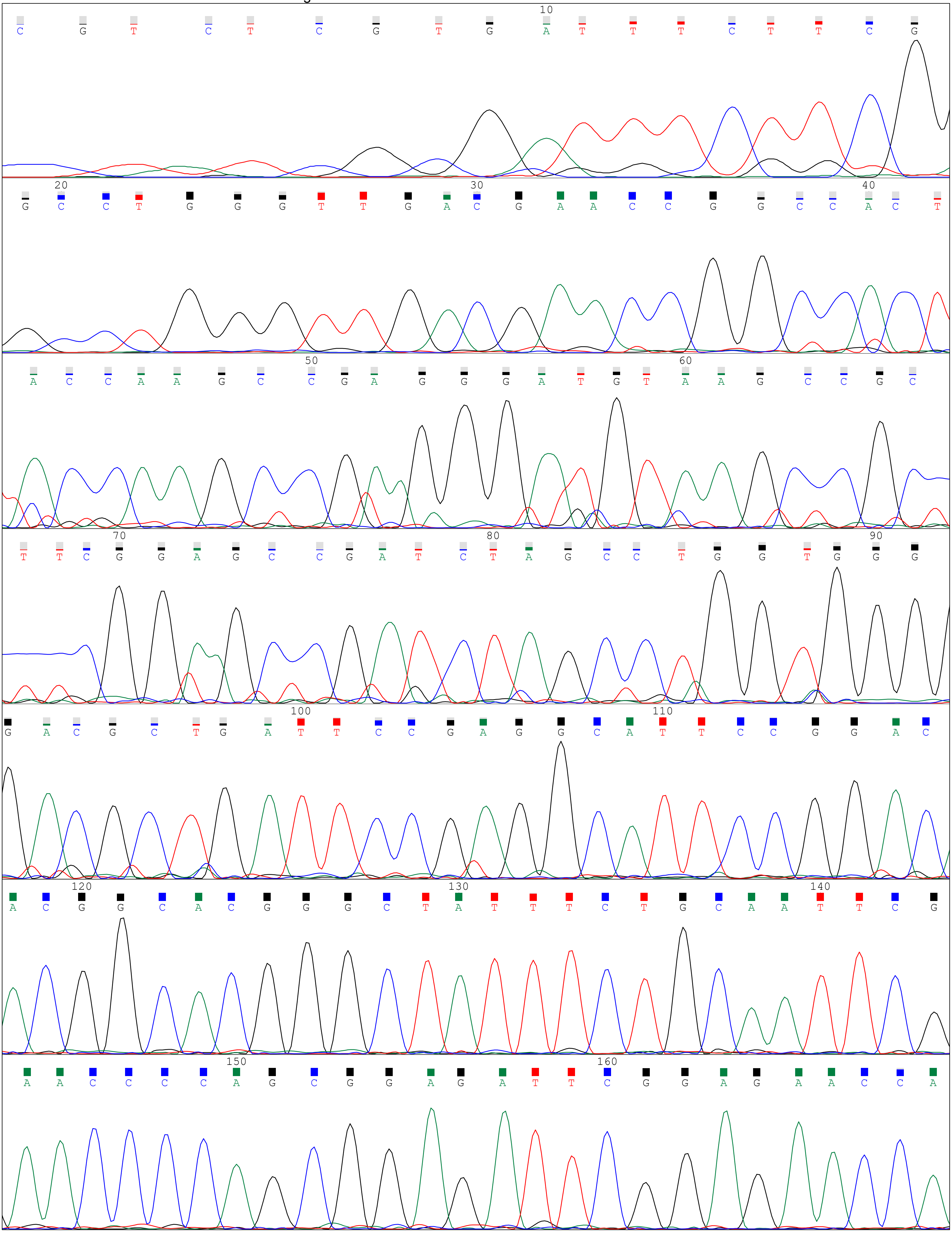

File: mutΔrifKatt1_cl2-LS172.ab1
Signal G:9720 A:11140 T:8657 C:16011
Sample: mutΔrifKatt1_cl2-LS172
301 bases in 3841 scans

Run Ended: Jan 7, 2021, 1:21:10
Comment: 2389291-I01-EmmanuelleDarbon-50-463277508-ED2
Lane: 7 Base spacing: 14.68

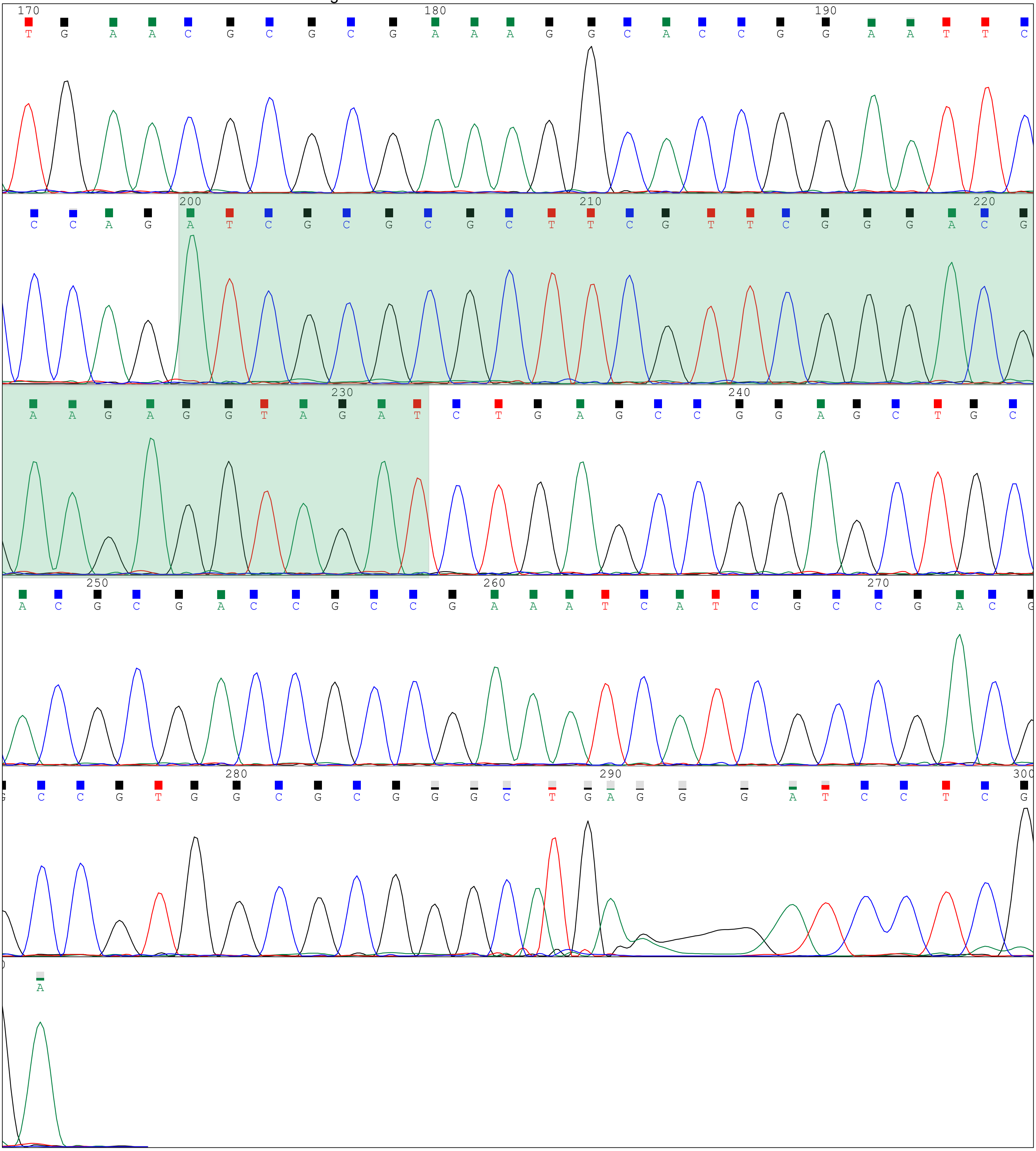

File: mutΔrifKatt1_cl3-LS172.ab1
Signal G:13771 A:15173 T:11163 C:19261
Sample: mutΔrifKatt1_cl3-LS172
309 bases in 3978 scans

Run Ended: Jan 7, 2021, 1:21:10
Comment: 2389291-A01-EmmanuelleDarbon-50-463277508-ED3
Lane: 15 Base spacing: 14.45

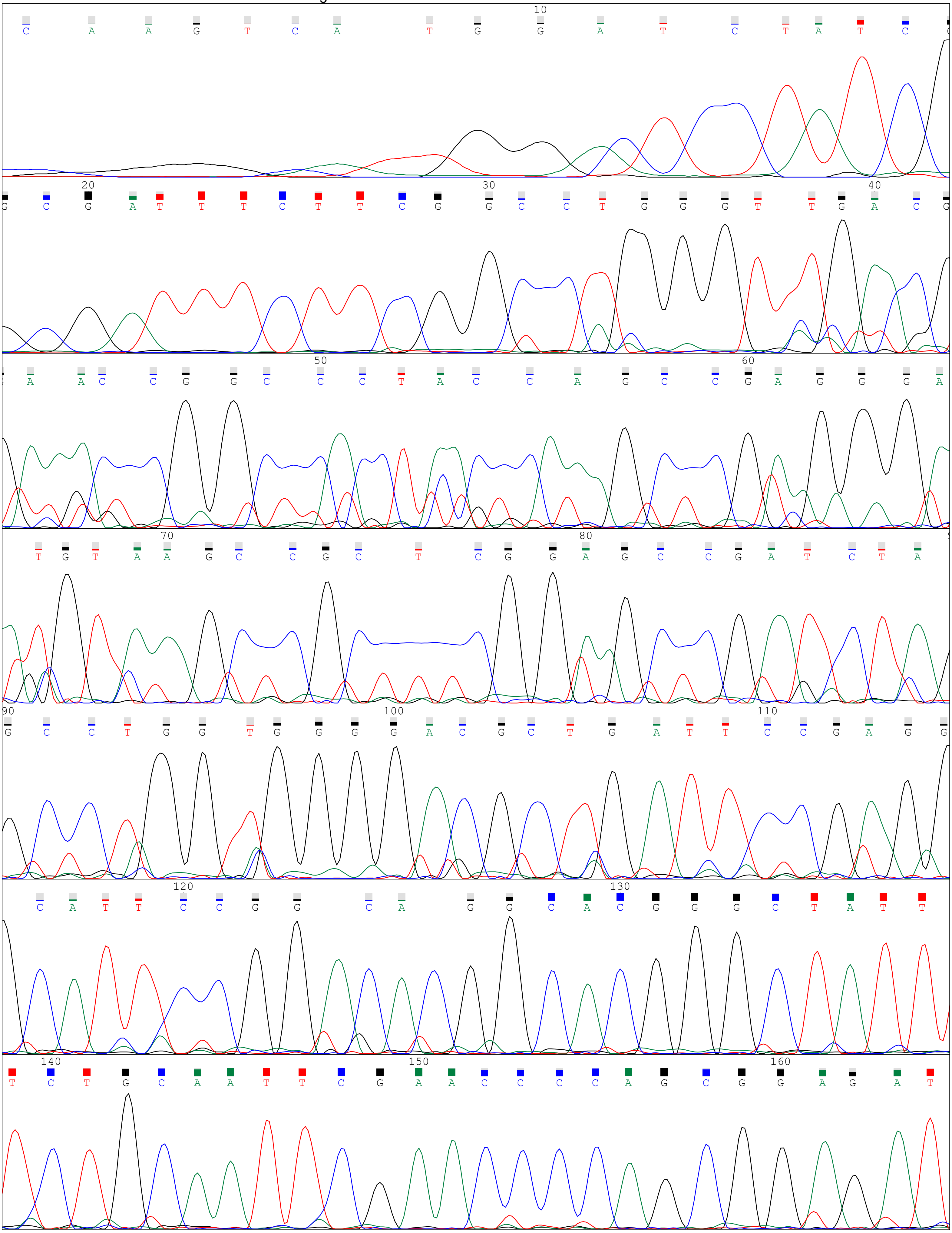

File: mutΔrifKatt1_cl3-LS172.ab1
Signal G:13771 A:15173 T:11163 C:19261
Sample: mutΔrifKatt1_cl3-LS172
309 bases in 3978 scans

Run Ended: Jan 7, 2021, 1:21:10
Comment: 2389291-A01-EmmanuelleDarbon-50-463277508-ED3
Lane: 15 Base spacing: 14.45

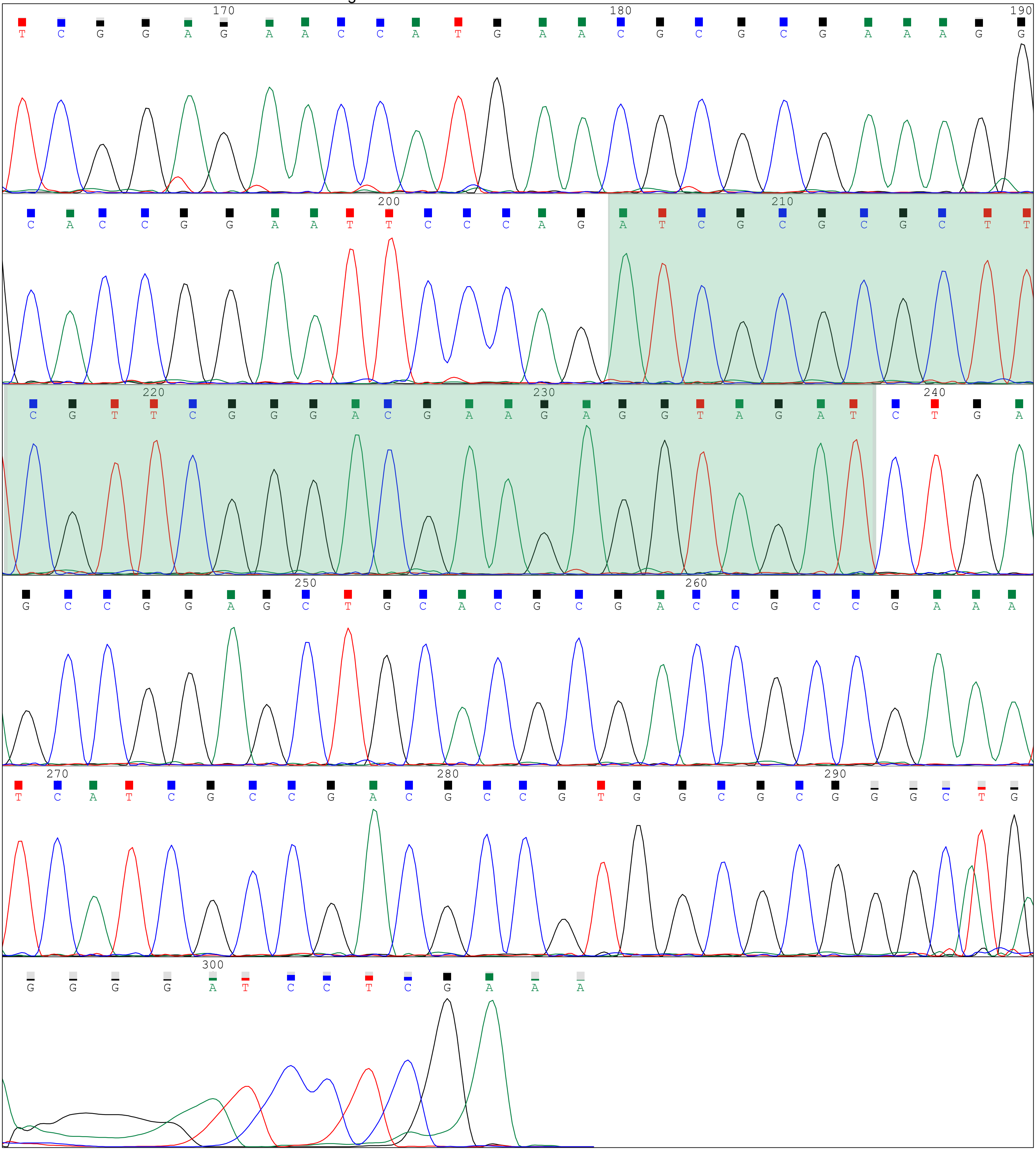

Supplement: Supplementary file 1 [file microorganisms-10-00828-s001.zip › File S1 - Sequence data of marker-free rifK cluster mutants.pdf]
