# Supplementary material for: Marker-Free Genome Engineering in Amycolatopsis Using the pSAM2 Site-Specific Recombination System
Source: Microorganisms. 2022 Apr 16;10(4):828. doi: 10.3390/microorganisms10040828 (PMC9033027; doi:10.3390/microorganisms10040828)

File S2: Sequence data of marker-free *rif* cluster mutants

Nucleotide sequence and chromatograms obtained after Sanger sequencing of three DSM 40773 Δ*rif::att1* independent clones are provided hereafter. The 33 bp scar expected to be formed after excision is highlighted in green. The high quality of the sequence shown in the chromatograms confirmed the homogeneity of the samples.

>mutΔrif-att1_cl1-LS220_I19.ab1

TTTTAAGTCAGTTCGCGAGTACCGCCGCAGGTCGGCCAGCGCCGCGCTGCGC
TCGGCAGCGGCCTGCGCCTCGGCCGGAGACGGCAGGCGCACCGGGGGCAAC
GCGGGGAAGAGCATCGACGTCGAGTTCGCACTCCTGGCACATGCGGGCCAG
TCTGGCAGGTCGAACTACCCACGTTGGTAATGGCTCCCACCGGATATCTACCT
CTTCGTCCCGAACGAAGCGCGCGATATCGTGGAAGGTGTTCTCGTAGGCCGG
TGACGCGGCGGCGGGGCCGGTGGTTCCCCGGGCGGTTGGGTGCGGTGGACG
GGAGA

>mutΔrif-att1_cl2-LS220_A19.ab1

CGCTCGGAATGGAGCGTCAGTTCGCGACGTACCGCCGCAGGTCGGCCAGCGC
CGCGCTGCGCTCGGCAGCGGCCTGCGCCTCGGCCGGAGACGGCAGGCGCAC
CGGGGGCAACGCGGGAAGAGCATCGACGTCGAGTTCGCACTCCTGGCACAT
GCGGGCCAGTCTGGCAGGTCGAACTACCCACGTTGGTAATGGCTCCCACCGG
ATATCTACCTCTTCGTCCCGAACGAAGCGCGCGATATCGTGGAAGGTGTTCTC
GTAGGCCGGTGACGCGGCGGCGGGGCCGGTGGTTCCCCGGGCGGTTGGGTGC
CGTGACGGGAGA

>mutΔrif-att1_cl3-LS220_G19.ab1

TGAAAAATCGTTCGCGACGTACCGCCGCAGGTCCGGCCCAGCCGCCGCCGCT
TGCGCTCGGCAGCGGCCTGCGCTCTCGGCCCGGAGAACGGCAGGCGCACCG
GGGGCAACGCGGGAAGAGCATCGACGTCGAGTTCGCACTCCTGGCACATGC
GGGCCAGTCTGGCAGGTCGAACTACCCACGTTGGTAATGGCTCCCACCGGAT
ATCTACCTCTTCGTCCCGAACGAAGCGCGCGATATCGTGGAAGGTGTTCTCGT
AGGCCGGTGACGCGGCGGCGGGGCCGGTGGTTCCCCGGGCGGTTGGGTGCG
GGGGACGGGAGA

File: mutΔrif-att1_cl1-LS220.ab1
Signal G:7320 A:6523 T:5924 C:16877
Sample: mutΔrif-att1_cl1-LS220
315 bases in 3937 scans

Run Ended: Apr 8, 2021, 14:49:21
Comment: 2424358-I19-EmmanuelleDarbon-50-502973328-ED1
Lane: 72 Base spacing: 15.06

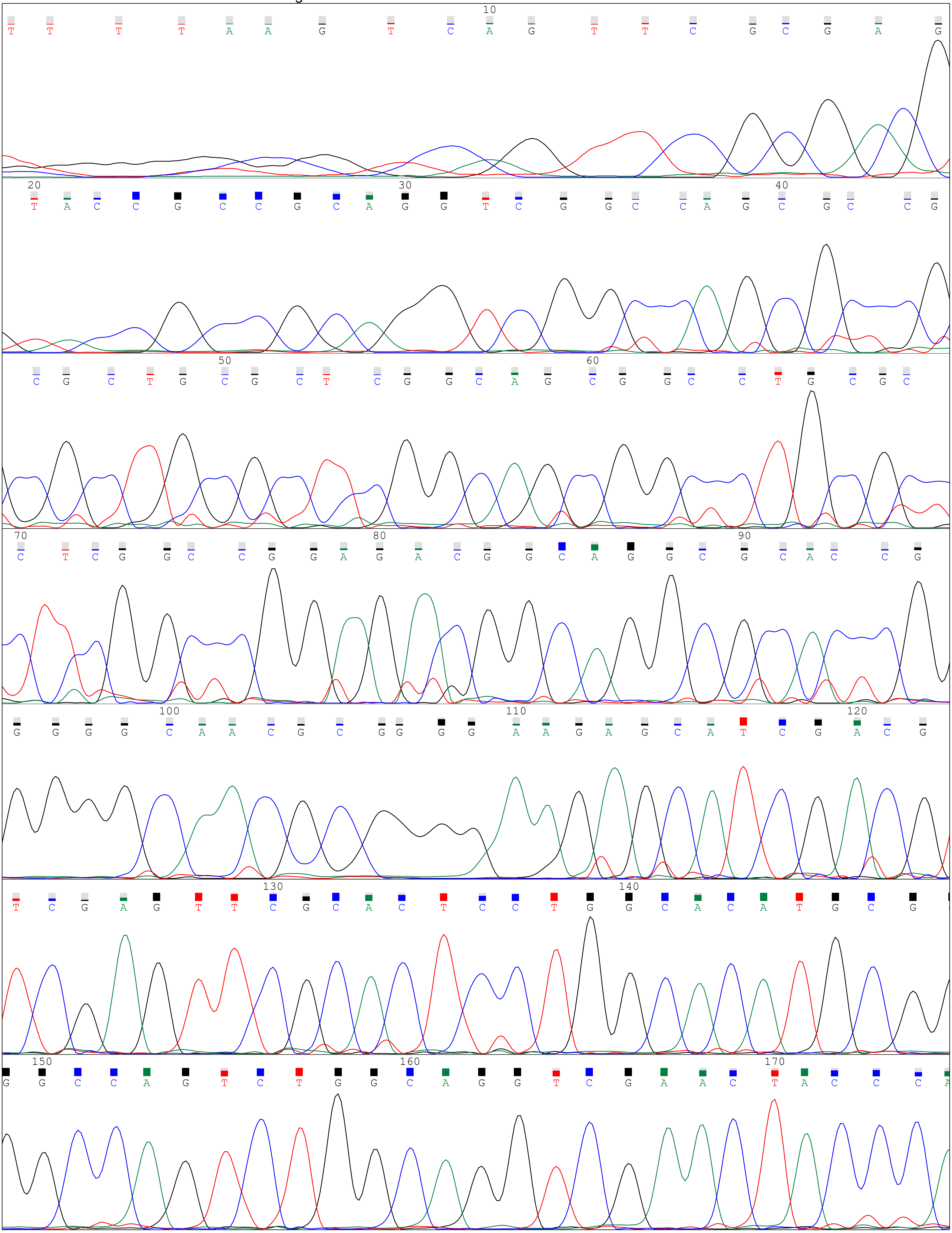

File: mutΔrif-att1_cl1-LS220.ab1
Signal G:7320 A:6523 T:5924 C:16877
Sample: mutΔrif-att1_cl1-LS220
315 bases in 3937 scans

Run Ended: Apr 8, 2021, 14:49:21
Comment: 2424358-I19-EmmanuelleDarbon-50-502973328-ED1
Lane: 72 Base spacing: 15.06

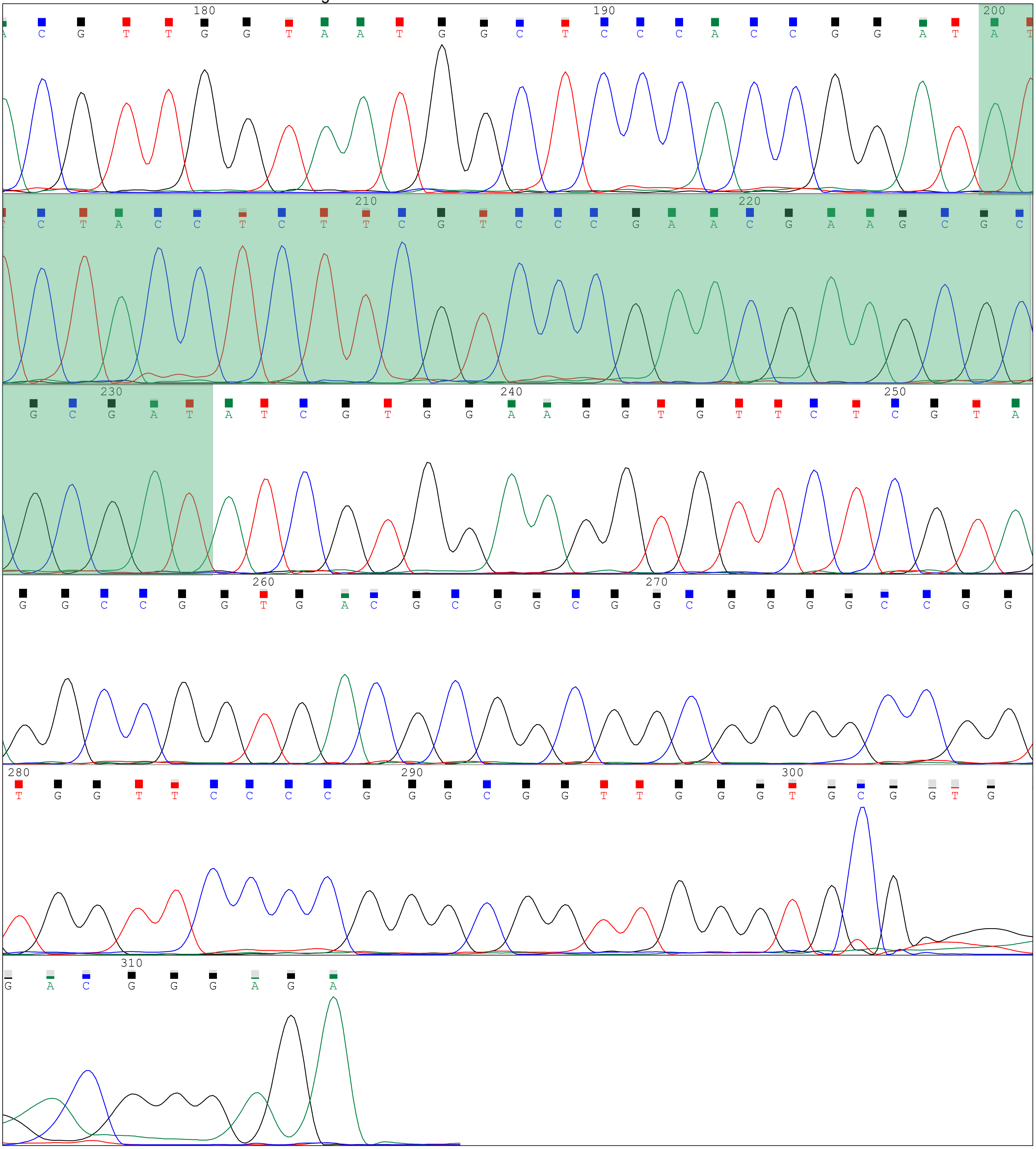

File: mutΔrif-att1_cl2-LS220.ab1
Signal G:1977 A:1760 T:1730 C:4825
Sample: mutΔrif-att1_cl2-LS220
323 bases in 3951 scans

Run Ended: Apr 9, 2021, 1:33:59
Comment: 2425017-A19-EmmanuelleDarbon-50-506386747-ED3
Lane: 80 Base spacing: 16.08

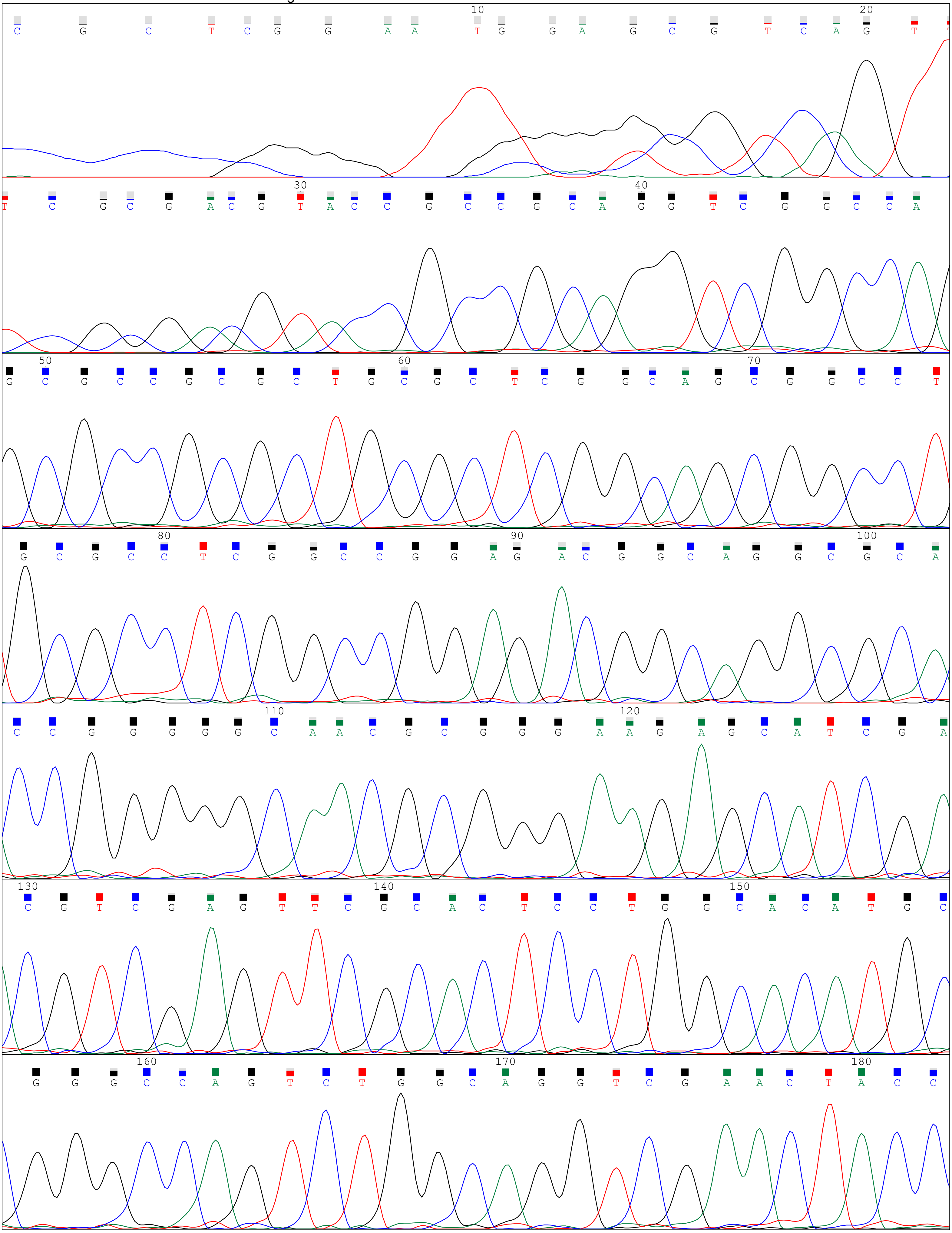

File: mutΔrif-att1_cl2-LS220.ab1
Signal G:1977 A:1760 T:1730 C:4825
Sample: mutΔrif-att1_cl2-LS220
323 bases in 3951 scans

Run Ended: Apr 9, 2021, 1:33:59
Comment: 2425017-A19-EmmanuelleDarbon-50-506386747-ED3
Lane: 80 Base spacing: 16.08

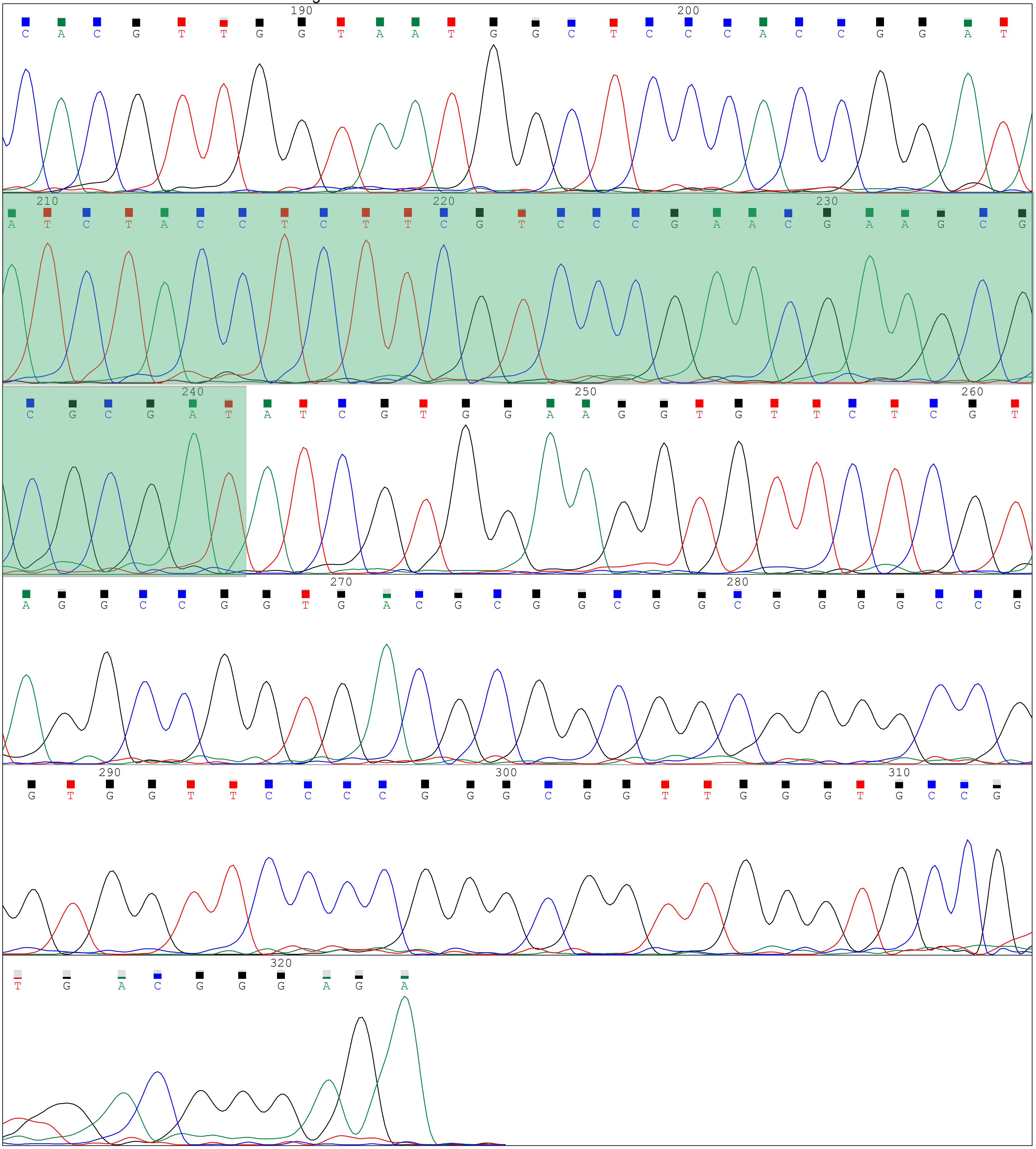

File: mutΔrif-att1_cl3-LS220.ab1
Signal G:10579 A:8710 T:8896 C:18815
Sample: mutΔrif-att1_cl3-LS220
322 bases in 3935 scans

Run Ended: Apr 8, 2021, 14:49:21
Comment: 2424358-G19-EmmanuelleDarbon-50-502973328-ED5
Lane: 74 Base spacing: 15.11

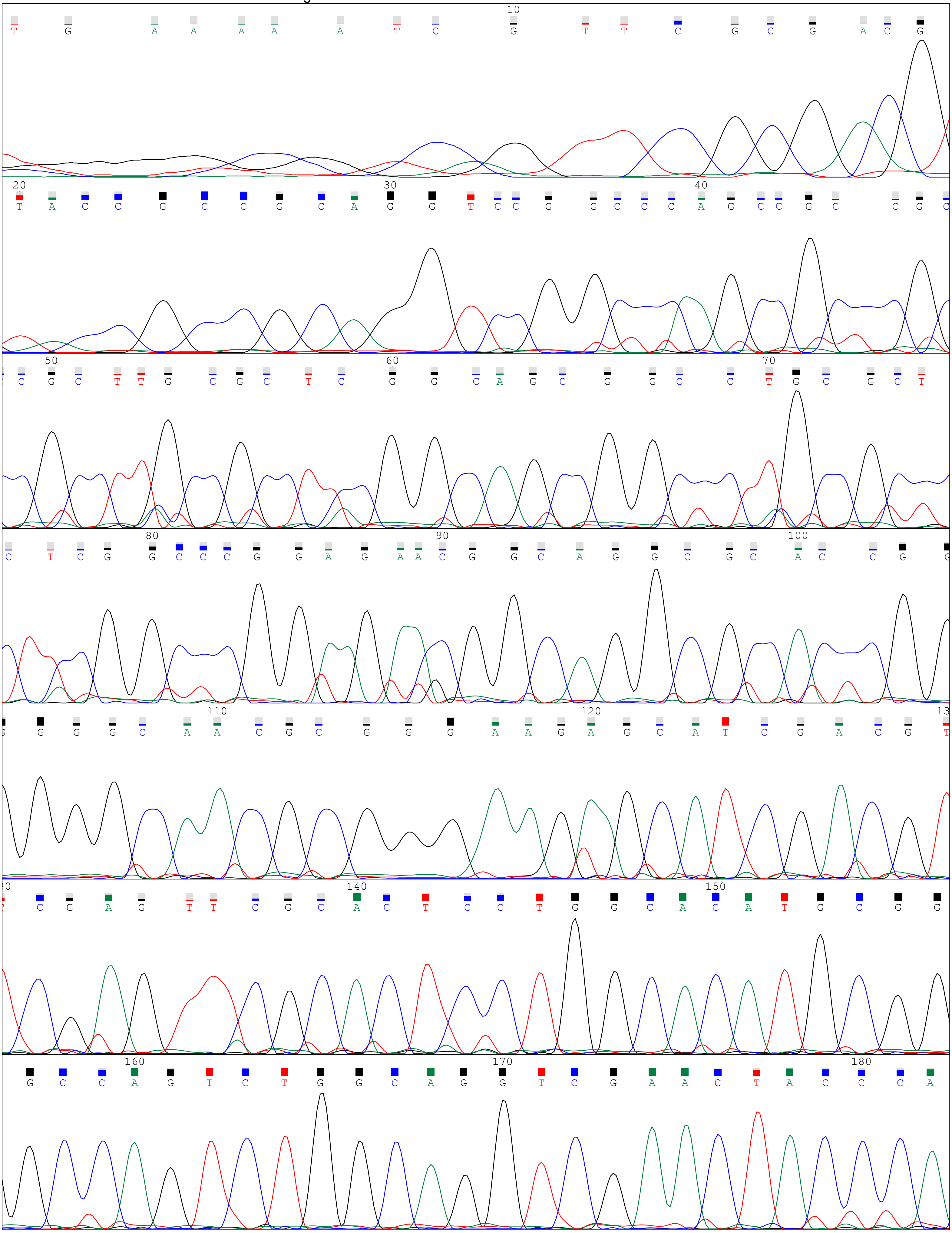

File: mutΔrif-att1_cl3-LS220.ab1
Signal G:10579 A:8710 T:8896 C:18815
Sample: mutΔrif-att1_cl3-LS220
322 bases in 3935 scans

Run Ended: Apr 8, 2021, 14:49:21
Comment: 2424358-G19-EmmanuelleDarbon-50-502973328-ED5
Lane: 74 Base spacing: 15.11

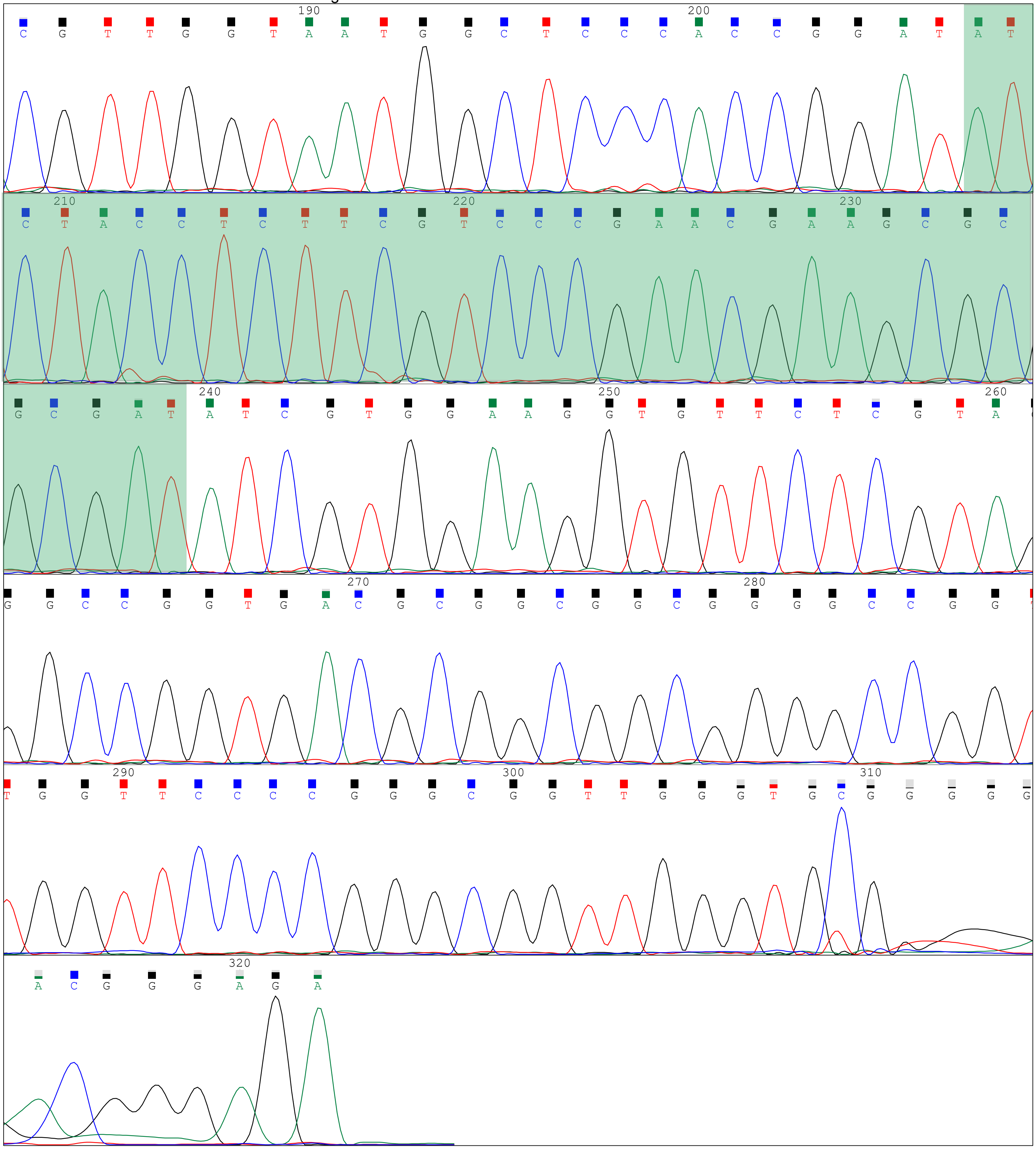

Supplement: Supplementary file 1 [file microorganisms-10-00828-s001.zip › File S2 - Sequence data of marker-free rif cluster mutants.pdf]
