# Supplementary material for: Marker-Free Genome Engineering in Amycolatopsis Using the pSAM2 Site-Specific Recombination System
Source: Microorganisms. 2022 Apr 16;10(4):828. doi: 10.3390/microorganisms10040828 (PMC9033027; doi:10.3390/microorganisms10040828)
Supplement: Supplementary file 1 [file microorganisms-10-00828-s001.zip › Table S1. Oligonucleotides used in this study.pdf]

Table S1. Oligonucleotides used in this study.

Name	Sequence§	Description
EDM79	TCCGTCTTGGGCTCAACCT	Sequencing of pEA01
EDM80	GTTAGCGGCGTGGAAGCGTC	Sequencing of pEA01
EDM81	ATCGGGTGGCTAGGTGAAGA	Sequencing of pEA01
EDM82	GAGCGCCTGATGCGGTATTT	Sequencing of pEA01
EDM83	GAGCAAGGTGAGATGACAGG	Sequencing of pEA01
EDM84	TGATGACCTGGCCGCTGTTC	Sequencing of pEA01
EDM85	GTGAAGCACAGCTCGACCAT	Sequencing of pEA01
EDM86	CAGGACTTCCTGGGCCGACT	Sequencing of pEA01
EDM87	TTTGCGCGTCGGGCGCGTCC	Sequencing of pEA01
EDM88	CAGATAGCCCAGTAGCTGAC	Sequencing of pEA01
EDM89	GCGCGGACGCGGTCTGTGGA	Sequencing of pEA01
EDM90	CACGTGGACACCGCAGGGAC	Sequencing of pEA01
EDM101	AGAGTTGGTAGCTCTTGATC	Sequencing of pEA01
EDM102	GGCTGGTTGTCAGTGATCGA	Sequencing of pEA01
EDM103	CCCGCAGCGCCCGACCGAAA	Sequencing of pEA01
EDM104	TGCCGTCCATGACCACAGCG	Sequencing of pEA01
EDM105	AGACCGTAGGCAAGCCAGTC	Sequencing of pEA01
EDM106	CGCTCACAATTCCACACATT	Sequencing of pEA01
EDM107	ACTGGCTTTCTACGTGTTCC	Sequencing of pEA01
EDM108	GACATCGGCAAGGTGTGGGT	Sequencing of pEA01
LC18	CGACATCAACCTCTGATTCC	Sequencing of pEA01
LS148	TTGCCACCGCGCTCATCAATC	Sequencing of pEA01
LS149	ACTGGAAAGCGGGCAGTGAG	Sequencing of pEA02
LS150	ATTTCAGTGCAATTTATCTCTTC	Sequencing of pEA03
LS151	GGATCCTGGCGCGGATGGGCGAAG	Amplification of the upstream homologous arm of *rifK*
LS152	CAGCTGGGAATTCCGGTGCCTTTCG	
LS153	CAGCTGAGCCGGAGCTGCACGCGACC	Amplification of the downstream homologous arm of *rifK*
LS154	AAGCTTGCAGCTCGCCGATGTCGTCC	
LS155	GCGGCCGCGATCACACCGACGATGGAG	Amplification of the upstream homologous arm of the *rif* cluster
LS156	GATATCCGGTGGGAGCCATTACCAAC	
LS157	GATATCGTGGAAGGTGTTCTCGTAGG	Amplification of the downstream homologous arm of the *rif* cluster
LS158	ATGCATCAGCTATGAGCGACTGTCAAGG	
LS161	CTGCAGATCTCCCGGCTCGTC	Couple of primers used to amplify the upstream junction created by replacement of *rifK* by the att1 *hyg* cassette (PCR 1 Figure 2)
LS182	GGAGGAGACCGCACGGTTG	
LS188	CGCCCGTGGGAAGGTGAGTGTC	Couple of primers used to amplify the downstream junction formed by replacement of *rifK* by the att1 *hyg* cassette (PCR 2 Figure 2)
LS189	GCGAGGTCGTCCTCGGCACTG	
LS172	GACGTCCATTACGGCAGCCA	Couple of primers used to verify the excision of the hygromycin cassette (PCR 3 Figure 2)
LS173	CGCTCATGCCCGCGCCACG	
LS71	GAGAAGCACACTGGCCCAAG	Couple of primer used to verify the integration of pRIF12 upstream of the *rif* cluster (PCR 1 Figure 3)
LS190	CGCACCGACACGAAGAAGAC	
LS191	CACTGGAACGACTGGATCTG	Couple of primer used to verify the integration of pRIF14 downstream of the *rif* cluster (PCR 2 Figure 3)
LS70	CGCGATAGTCACGCAGATAG	
LS220	AGCGTGGGAGCAGCGTAAGC	Couple of primers used to verify the excision of the *rif* cluster (PCR 3 Figure 3)
LS221	CTCCCGTCACGGCACCCAAC	

§Restriction sites added and used for cloning are underlined.
